# Participatory co-learning for human–wildlife coexistence: Reflections on a novel program applying systems thinking, nonviolent communication, and learning-based approaches

**DOI:** 10.1007/s13280-024-02032-5

**Published:** 2024-05-16

**Authors:** Ruth Kansky, Tarek Maassarani, Joern Fischer

**Affiliations:** 1 Department of Conservation Ecology and Entomology, Stellenbosch University, Private Bag X1, Matieland, 7602 South Africa; 2 Justice and Peace Studies, Georgetown University, Washington, DC 20057 USA; 3 Social-Ecological Systems Institute (SESI), Faculty of Sustainability, Leuphana Universitaet Lueneburg, Universitaetsallee 1, 21335 Lüneburg, Germany

**Keywords:** Collaborative governance, Human–wildlife conflict and coexistence, Learning-based approach in natural resource management, Nonviolent communication, Systems thinking, Wildlife governance

## Abstract

As wildlife habitats become increasingly fragmented, sharing landscapes with wildlife is becoming difficult and complex. Because stakeholders with diverging interests struggle to collaborate to manage human–wildlife interactions, new approaches are needed. Here we reflect on a novel participatory learning program we implemented with farmers in communal conservancies in the Zambezi region of Namibia. The 9 week program aimed to understand why human–wildlife conflict remained a challenge. We combined three theoretical framings in the program design—systems thinking, nonviolent communication, and learning based approaches. We summarize key outcomes of each session and reflect on the overall program. We found a synergistic effect of the three framings and concluded that our integrated program had been a useful collaborative learning tool to understand the human–wildlife governance system, identify interventions, empower communities, and build capacity for collaboration to improve human wellbeing and human–wildlife interactions. Drawing on our experience, we make suggestions for how the program could be adapted for similar or other environmental problems elsewhere.

**Supplementary Information:**

The online version contains supplementary material available at 10.1007/s13280-024-02032-5.

## Introduction

Human wildlife conflict (HWC), human–wildlife coexistence, human–wildlife interactions, biodiversity conflicts, and most recently, convivial conservation (Fletcher and Toncheva 2021) are varying concepts with different framings but are commonly applied to situations in which people and wildlife struggle to share overlapping areas (Pooley et al. 2021; Bhatia et al. 2019). The diversity of terminologies and framings of the issue reflects the emerging realization of the complexity of the problem. Initial research in the field of HWC saw the problem as “tame”—mitigation measures could be implemented to prevent or reduce the damage from wildlife and the problem would be solved. However, the complexity of the field is now emerging with the understanding that in addition to negative impacts there are also complex human relationships and governance issues to manage. Along with this realization are calls for more transdisciplinary methods (Hartel et al. 2019) and a more diverse toolbox. Among these new tools are participatory processes such as conflict transformation (Madden and McQuinn 2014; Zimmerman et al. 2020), participatory dialogues (Redpath et al. 2017; Sterling et al. 2017; Salvatori et al. 2020; Marino et al. 2021), scenario planning (Konig et al. 2020; Jiren et al. 2021), and citizen science (Ostermann-Miyashita et al. 2021).

**Fig. 1:**
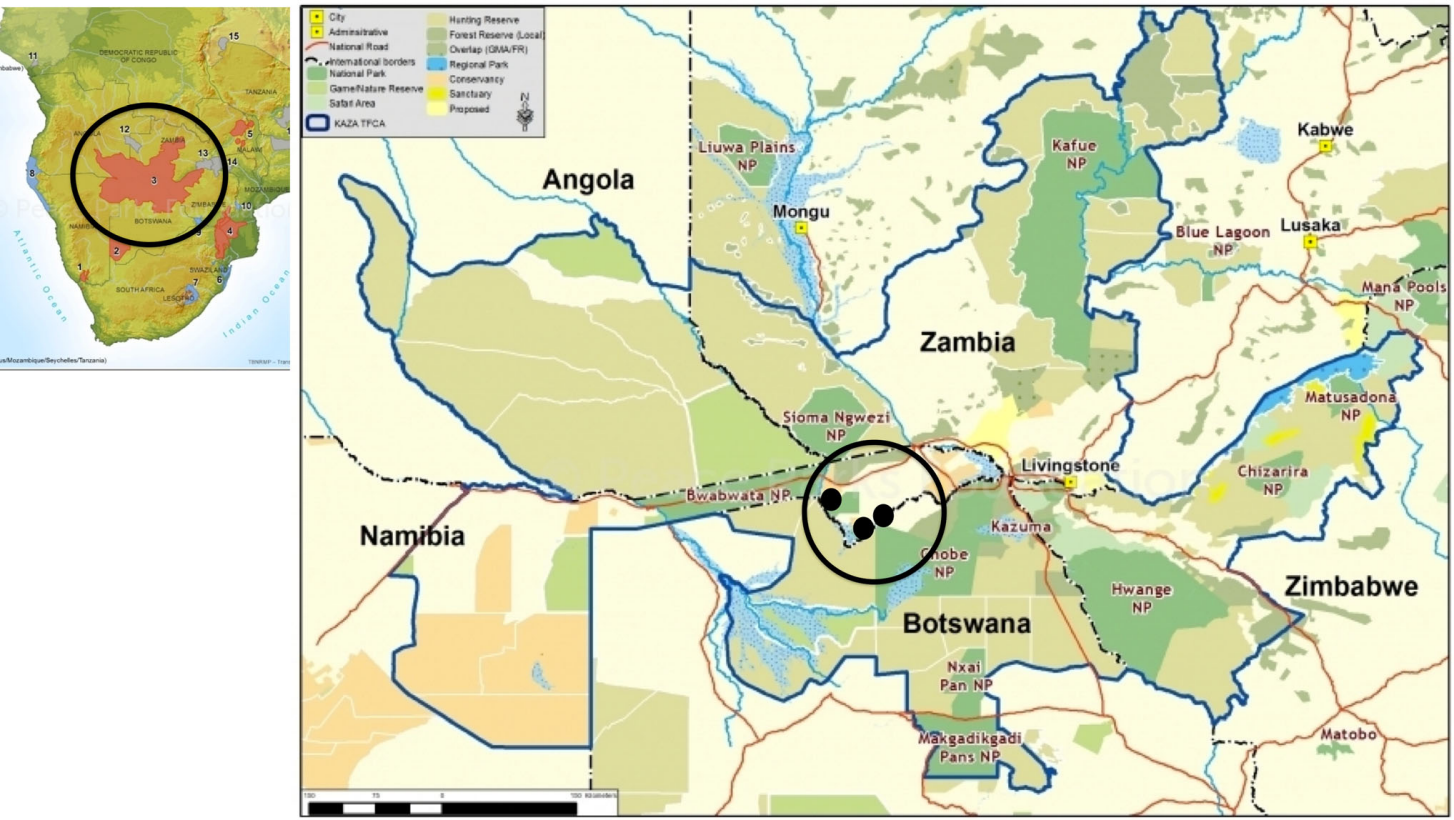
Map of the study area—the Kavango–Zambezi Transfrontier conservation area in southern Africa (blue outline), showing the Mudumu complex in the Zambezi region of Namibia. Conservancy locations are indicated in black dots. *Source*: Map courtesy of NACSO

Viewing human–wildlife relationships as occurring within complex social–ecological systems (Ceauşu et al. 2019; Balasubramaniam et al. 2021), we developed and implemented a novel participatory learning process to promote human–wildlife coexistence in conservancies in the Zambezi region of Namibia that experience high levels of human–wildlife conflict and form part of the Kavango–Zambezi Transfrontier Conservation Area (Fig. 1). Transfrontier Conservation Areas (TCA) are an initiative of the Southern African Development Community (SADC) that aim to conserve the rich wildlife of the region as well as implement projects to improve human livelihoods (Hanks 2003). A TCA is a large ecological region that straddles the boundaries of two or more countries and encompasses one or more protected areas, as well as multiple resource use areas (SADC 1999). In such landscapes, the negative wildlife impacts on people can be significant (Nyhus et al. 2004; Sinthumule 2016; Salerno et al. 2020; Stoldt et al. 2020) and a key challenge is to identify viable options for human–wildlife coexistence (Crespin and Simonetti 2019; Stoldt et al. 2020; Thondhlana et al. 2020)—which we define as the willingness of communities to share the landscape with, and tolerate the potential costs from wildlife, while ensuring sustainable wildlife populations. Community-Based Natural Resource Management (CBNRM) is a widely used collaborative governance approach in the region that aims to improve livelihoods and offset the costs of disserves from wildlife (Nuulimba and Taylor 2015) through providing benefits from sustainable commercial and subsistence hunting and tourism. These benefits are presumed to promote human–wildlife coexistence. Namibia’s CBNRM program is considered one of the more successful initiatives in Southern Africa (Nelson and Agrawal 2008; Nuulimba and Taylor 2015; MET/NACSO 2018), but HWC remains a key challenge with concern that it could undermine the long-term acceptance and hence sustainability of the program (Salerno et al. 2020; Jiren et al. 2021).

Given that human–wildlife coexistence challenges remain in one of the more successful CBNRM programs in Africa, we used conservancies in the Zambezi region of Namibia as a case study to implement a new type of learning program. The general program goal was to improve human–wildlife coexistence through better understanding how HWC is managed within the conservancy governance system and to understand why HWC remains a challenge after 30 years of CBNRM in Namibia.

The design of our learning program was informed by three elements, namely systems thinking, nonviolent communication, and learning-based approaches. First, systems thinking is an orientation or a perspective that is used to better understand “wicked” problems (Muro and Jeffrey 2008). Systems thinking helps people see the world as a series of interconnected and interdependent parts rather than approaching different parts in isolation (Meadows 2008). It encourages the search for patterns of interaction and underlying structures that shape the emergent patterns of systems behavior. In natural resource management, systems thinking is widely believed to be critical for institutional innovation and transitions to resilient and sustainable futures because it can help to navigate growing complexity facing the world (Meadows 2008; Berkes 2009; Arnold and Wade 2015; Wals 2015; Wahl 2016; Fischer and Riechers 2019). However, in the context of human–wildlife interactions, this framing is still new, with many interventions continuing to use a linear logic where implementing technological solutions is expected to solve the problem. Such linear thinking masks underlying complexities, for example, when different stakeholders disagree on the best approaches (Lute and Attari 2017; 2018; Jiren et al. 2021), when there is a history of underlying social conflicts (Manfredo 2008; Jacobsen and Linnell 2016; Rust et al. 2016; Zimmerman et al. 2020), when accountability is lacking, or when the governance system does not fit the local context (Ravenelle and Nyhus 2017; Kansky 2022).

Second, nonviolent communication (NVC) or compassionate communication (Rosenberg 2005; www.cnvc.org) is a practical form of awareness about oneself and others, inspired by Gandhi’s philosophy of non-violence. The underlying assumption is that meeting universal needs is key to human well-being (Max Neef et al. 1989; Tay and Diener 2011; Jolibert et al. 2014), while human behaviors are merely strategies employed to meet these basic needs. According to this framing, conflict results because one party uses strategies that prevent another party from meeting their needs. War and violence are extreme strategies to meet one’s needs when all hope is lost. Since all humans are motivated by the same human needs, in conflict mediation, awareness of needs allows individuals to understand themselves and each other without resorting to divisive interpretations or judgments, and this brings a quality of compassion, non-judgment, honest expression, trust, and equity to the connection with oneself and to others. The intention of many non-violent communication practitioners is to facilitate a socio-linguistic transformation of domination systems (based on coercion and power-over) into partnership systems (based on willingness and power-with). NVC has rarely been applied in the context of HWC (but see Kansky and Maassarani 2022), and we predicted it to be particularly useful in bringing equality and trust to learning processes, two factors that are recognized as being especially important to facilitate learning (Newig et al. 2019; Armitage et al. 2012). In addition, we also hypothesized that applying the concepts of universal needs and empathy to wildlife could increase tolerance as in our previous studies empathy was found to be a significant driver of tolerance (Kansky and Kidd 2024).

Third, learning-based approaches are seen by many as essential in participatory decision-making, adaptive management (Rist et al. 2013), collaborative governance, and sustainability transitions (Pahl-Wostl 2009; Berkes 2017). This is because they have the potential to mitigate the complexity, uncertainty, and conflict characteristic of natural resource management and sustainability problems by encouraging experimentation, innovation, and adaptability (Baird et al. 2014; Bostrom et al. 2018). Through their participatory and collaborative framing, they also provide spaces for democratic engagement (Wals 2015). Participatory and dialogue processes are promoted to build trust and collaboration in best practice by the IUCN HWC task force (IUCN 2023). However, a focus on learning is not explicit, and case studies are still rare in the context of human–wildlife interactions.

In Kansky and Maassarani (2022), we reported the results of our learning program specifically with respect to nonviolent communication outcomes. In Kansky (2022), we focused on the specifics of the local human–wildlife system. In the current paper, we do not repeat these earlier, detailed findings, but rather, we focus on the learning-based approach element of the program as well as on the effectiveness of combining all three elements (systems thinking, Nonviolent Communication, learning-based approaches). To that end, we reflect on the learning program as a whole, summarizing the results for each week of the program. Drawing on the overall experience we thus gained, we make suggestions for how the program could be adapted for similar or other environmental problems elsewhere.

## Theoretical foundations

### Learning-based approaches

Learning in natural resource management and governance is a growing area of research but still lacks conceptual clarity and consensus on how to define terms, the factors that determine learning processes and outcomes, what the learning outcomes are or should be, and how to measure or evaluate outcomes and impacts (Suskevics et al. 2018; Ernst 2019; Newig et al. 2019; Van Epp and Garside 2019; Van Poeck et al. 2020). Despite this lack of consensus, recent reviews are making progress synthesizing this body of research (Suskevics et al. 2018; Newig et al. 2019) and three broad conceptualizations of learning are emerging. Firstly, *transformative learning* (Mezirow 1991) considers the process of learning as individuals evaluate and refine their attitudes, values, worldviews, and frames of reference as well as individual actions (Suskevics et al. 2018). Secondly, *social learning* (Reed et al. 2010) is the most widely used, though different conceptualizations are evident in the literature. One that is often used is by Reed et al. (2010): a change in understanding that goes beyond the individual to become situated within wider social units or communities of practice through social interactions between actors within social networks. The emphasis here is on collaborative and multi-loop learning processes (Argyris and Schon 1996) that lead to changes in understanding of a topic as well as changes in relationships at individual and collective levels and sometimes to adaptive or transformative change (Suskevics et al. 2018). Thirdly, *policy learning* is a term broadly used to describe acquiring new knowledge to inform policy and policymaking (Cairney 2012) and seeks to understand policy outcomes by engaging state officials, policy networks, or policy communities (Suskevics et al. 2018). Thus, the three concepts differ based on their assumptions about how learning is linked to change outcomes (Suskevics et al. 2018). When combined with systems thinking, collaborative learning and dialogue processes can be used to unpack a “system of interest” by focusing attention on processes, relationships and interactions between the system’s components as seen by different stakeholders (Dyball et al. 2009).

## Materials and methods

### Case study: Human–wildlife coexistence learning program

#### Study area

We focused on communities in the Kwando Wildlife Dispersal Area of the Kavango–Zambezi Transfrontier Conservation Area in Namibia (Fig. 1). The area is bordered by the Kwando, Linyanti, Chobe, and Zambezi Rivers and is a region of woodlands, swamps, and flood plains. There are three national parks in the landscape, Babwata, Mudumu, and Nkasa Lupala, as well as the Zambezi State Forest. Communal lands and 15 conservancies surround these protected areas. Conservancies are communal lands that are unfenced, multiple use areas with defined boundaries and that serve as wildlife corridors within the regional context. Conservancy governance is guided by national policies promulgated by the Ministry of Environment Forestry and Tourism (MEFT), and local policies that are implemented through elected and salaried community members who serve on Conservancy Management Committees. These committees, in turn, collect and distribute revenue generated from trophy hunting and tourist lodges (Nuulimba and Taylor 2015; MET/NACSO 2018). The study area we focused on encompassed three conservancies between Nkasa Lupala and Mudumu National Parks: Bamunu, Balyerwa, and Mayuni (Fig. 1). One of the most common threats to livelihoods is human–wildlife conflicts (Glatz-Jorde et al. 2014; Salerno et al. 2020; Stoldt et al. 2020). For example, Kansky (unpublished) reported 67% of respondents in the Zambezi region experienced damage from wildlife with the mean yearly amount of damage of 677 USD, while Stoldt et al. (2020) reported that 60% of surveyed community members in the region felt that HWC had increased in recent years. In addition, Salerno et al. (2020) reported that higher perceptions of crop damage were associated with higher food insecurity.

### Program design

Using the theoretical foundations described above, we developed the Human–Wildlife Coexistence Social Learning Program. The systems thinking component informed our idea of “unpacking” the governance system in the conservancies with a focus on how it facilitates or hinders human–wildlife coexistence. The learning aspect informed our idea of recruiting change leaders from the community and then allowing a better understanding of and improvements to the governance systems to emerge through group discussions among themselves and with invited guests. The nonviolent communication training facilitated constructive dialogues, creating an atmosphere where participants could feel safe and be heard with respect and equality, and bring clarity and understanding to the dialogues. The new communication skills and increased empathy between people and toward wildlife would promote human collaboration and increase tolerance toward wildlife. In previous research applying the Wildlife Tolerance Model, empathy was identified as a significant driver promoting tolerance to damage-causing wildlife (Kansky et al. 2021; Marino et al. 2021; Kansky and Kidd 2024).

The program consisted of 11 weeks of half-day workshops once a week and took place between April and August 2019. For the first four sessions, the program consisted of training in Nonviolent communication that was integrated into dialogues on topics related to living with wildlife. The discussion topics were: 1. Good and bad aspects of living with wildlife; 2. Present and future perceptions, ideas and wants around living in conservancies with wildlife; 3. Cultural stories about wildlife and a role-play discussion with an elephant; 4. Negative experiences with conservancy or MEFT personnel. For sessions 5–8, guests were invited to the workshops where participants had the opportunity to ask them questions. Participants decided who to invite and these were generally members from the conservancy management committee such as the field officer (head of game guards), managers, enterprise officer, area representatives, chairperson, vice chairperson, or advisors. In addition to the invited guests in sessions 5–8, the first 1.5 h before the arrival of guests was spent as follows. For session 5–6, participants formed working groups to prepare presentations to the group on existing policy documents (constitution, national HWC policy, zonation plan, conservancy HWC management plan) followed by a discussion about the policies. For sessions 7–8, time was spent on review and practice of NVC. Session 9 involved an NVC test, discussion around project ideas, and closing ceremony with presentation of certificates.

After the first 9-week program, participants were invited to return after a one month break for another two sessions to design a HWC mitigation project using a log frame model. The first session involved defining problems while the second session involved building a theory of change and populating the log frame with activities. We do not, however, report on these extra two session in the current paper.

Throughout the program, each workshop started with a “check-in” session where participants were invited to verbally reflect on the previous week’s session. They were asked: “I would like to hear from some of you what, if anything, you learned from last week and what if anything changed in the way you think or behave as a result of attending the workshop.” If participants mentioned a change in thinking or behavior the facilitator asked them: “What do you think you would have done before attending the workshops?” Time permitting, a similar “check-out” session generally took place at the end of each session where participants were asked the same question regarding the current session. A detailed description of the aims and activities of each session is given in Table S1.

To recruit participants, conservancy management leaders were sent information about the upcoming program. Upon arrival of the authors in the area, community meetings were organized to explain the program and enlist volunteer participants. Criteria for participation were membership of a conservancy, being a farmer, interest in being a local change leader, preferable age between 20 and 40 years, and willingness to commit to at least 9 weeks of the 11-week program. We aimed to recruit 20 participants (10 men and 10 women) from each conservancy. All workshops were conducted in English by the two first authors of the paper, each author covering different sections. However, the second author who was from the USA and very experienced in facilitating workshops based on NVC and restorative justice conducted most of the sessions. The second facilitator was more experienced on the topic of human–wildlife conflict and coexistence. A translator from the region applied consecutive translation into the local siLozi language.

At the start of the program, participants filled in consent forms and made agreements on conduct during workshops. Consent was also requested and given to record the workshops. Ethical clearance was obtained from the SU ethics committee (ref. 0967).

### Data analysis

**Fig. 2:**
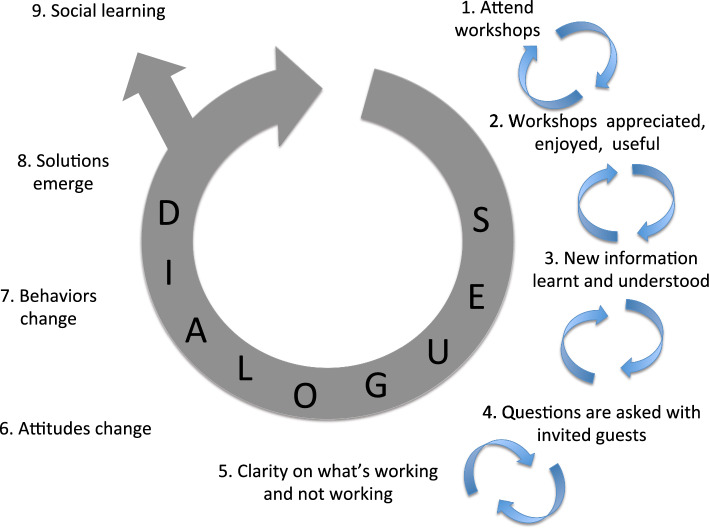
Theory of change showing the nine main codes

In Kansky (2022) and Kansky and Maassarani (2022), we gave a detailed description of the data analysis process and results with respect to Nonviolent communication and systems thinking, respectively. Briefly, to describe the program and its outcomes, we transcribed the workshop dialogues verbatim. We then used deductive, qualitative content analysis to construct a coding tree based on prior knowledge, the goal of the program, and our assumptions about the change process actuated by the program. Our hypotheses were that if participants attended the workshops and found them interesting and useful, they would appreciate the workshops, gain new knowledge, and understand the lessons. Through the dialogue process with invited guests, they would ask questions and understand what is working and not working in the conservancy. This would then result in changes in their attitudes and behavior, emergence of solutions, and ultimately sharing these with people in the broader community who did not attend the workshops (Fig. 2). In relation to the Nonviolent communication training, attitude and behavior changes would demonstrate increased empathic concern for both other people and wildlife. The coding tree categories are reported and explained in Table 1.

**Table 1:** Nine main codes, their definitions, and frequencies from the transcribed dialogues

	Main code	Freq	%
1.	*Problems*—records of what is not working well in conservancies	409	38.92
2.	*Knowledge and understanding*—records that showed new knowledge or better understanding of an issue or topic	148	14.08
3.	*Questions*—records of the questions that participants asked of invited guests	120	11.42
4.	*Appreciation*—records where participants expressed gratitude for the workshops or any specific component of the workshop	105	9.99
5.	*Working*—records of what is working in the conservancy	83	7.90
6.	*Behavior change*—records of actual changes in behavior, often compared to how the person would have behaved before attending the workshops	79	7.52
7.	*Attitude change*—records that reflected how a person’s thinking, beliefs, or intention to act toward a psychological object changed toward being more favourable (a psychological object being any discernible aspect of an individual’s world, including an object, a person, an issue, or a behavior (Fishbein et al. 2010)	44	4.19
8.	*Solutions*—records of ideas that participants suggested to solve specific problems	38	3.62
9.	*Social learning*—records where participants reported sharing ideas or knowledge with people not attending the workshops	25	2.38
	Total	1051	100.00

In the current paper, we primarily reflect on the results in relation to the theoretical framings of the study. For that reason, we only provide summaries of each session’s discussion categorized according to the three theoretical framings. In Supporting Information Tables S4–S13, we provide the frequency and some quotes for each of the main and subcategories for the reader to delve into the context more deeply. For those wishing to see more quotations, we refer the reader to the supplementary information in Kansky (2022) and Kansky and Maassarani (2022)—here, our focus is on evaluating the learning program as a whole rather than on detailing the specific challenges of the study system.

### Evaluation of impacts after three years

In July 2022 after three years since the program, participants were invited to come to an evaluation group meeting to share what they had been doing since the 2019 program and reflect on the medium-term impacts of the program. Summaries of the notes from this evaluation group meeting are reported in Table S3.

## Results

### Socio-demographic profile and attendance of participants

Fifty-nine community members initially signed up from the three conservancies; 54 attended; and > 80% of participants attended at least seven of the nine workshops (Table S4). The lowest number of participants that attended a session was 70% (Table S1). The average age of participants was 30.5 years. The average highest level of education was grade 10. The average number of adults per household was 2.8 and children 3.8. The average yearly total household income was 500 to 10,000 Namibian dollars ($38–$770 US) where 60% of households supplemented farming with other sources of income, mostly from government support grants and occasional jobs.

From the transcribed and coded dialogues, we identified 1051 dialogue sections that could be classified according to the nine main code categories (Table 1). Seven of the main codes could be divided into subcategories. In Tables S5–S13, we list the main codes and sub-categories and give an example of each. In Kansky (2022), the *working* and *problems* categories are discussed in detail, and in Kansky and Maassarani (2022) we focus on the Nonviolent communication training outcomes.

Summaries of the key outcomes of each workshop session for all the conservancies combined are reported in Table S1. These outcomes are based on the feedback sessions by participants at the start and end of each week. These are reported in three sections, based on the three theoretical elements on which the program was designed, namely systems thinking, learning-based approaches, and Nonviolent communication. Table S2 gives a more detailed account for each conservancy and includes the issues that emerged during the discussions in each session as well as detailed outcomes for each conservancy.

### Evaluation of impacts after three years

Of the 54 participants who attended the program, 32 (59%) came to the meeting. The remaining were either not reachable or unable to attend. Detailed summaries of the focus group discussions are given in Table S3, and five themes emerged from these discussions as follows:

1. *Application of mitigation measures*—Most participants reported applying mitigation measures in their fields and doing outreach and awareness with their family and neighbors around the need to apply them. This generally resulted in improved harvests.
2. *Application of knowledge of animal behavior during wildlife encounters*—Most reported using the information and felt it improved their safety.
3. *Social learning*—Most participants reported sharing their knowledge with their family or community, especially around mitigation measures and animal behavior. One group took a structured approach to the outreach in collaboration with game guards and using Nonviolent communication. They reported that game guards were grateful as it reduced their workload and farmers were appreciative as they were able to improve their yield. In this conservancy compensation, claims were reduced by 50%. They also reported sharing the information with others in their community, who showed appreciation for the knowledge. Other groups had tried to take a structured approach but were hampered by COVID and a lack of support from the conservancy.
4. *Empowerment and improved communication* through Nonviolent communication and empathy training. Those that reported personality changes from being ill tempered to more agreeable at the end of 2019 indicated those changes had persisted and were still having a positive impact on their lives. Through this many had been elected to leadership positions or believed, they had been successful in getting jobs through increased confidence and communication skills.
5. *Leadership*—Nine (28%) reported being elected to leadership positions, including in school boards, churches, advisors, a political party and as area representatives. Others reported being more involved in conservancy affairs, speaking out at meetings, or being consulted by the conservancy, although there were also reports of lack of support from conservancy, especially in setting up projects.

## Discussion

We designed a participatory learning program in conservancies in the Zambezi region of Namibia, a region with high human–wildlife conflict, to better understand how human wildlife coexistence is managed within the communal conservancy governance system that Namibia is well known for. The design of our program was based on thinking from three approaches, namely systems thinking, Nonviolent communication, and learning-based approaches. Each has their different but complementary philosophical foundations that overall proved useful.

The *systems thinking* element was useful to situate human–wildlife interactions as one element within the conservancy governance system. By interrogating the whole system, we were able to understand how different elements are linked and the assumptions behind how human–wildlife coexistence is presumed to emerge. We were able to achieve this through the rich conversations during the workshops where a wide variety of topics were covered based on the questions that participants asked the guests as well as during various training sessions (Tables S1, S2). For example, we learnt that information flow between conservancy management and the members was poor and that area representatives who were elected by their community to represent them were a key bottleneck in the system, primarily because they lacked the skills and motivation to report back to their community. They also did not provide information back to the management from the community (see Kansky 2022). In a forthcoming publication, we identified 35 elements (variables) that are important components of the social and ecological system and show how they interact using causal loop diagrams (Kansky forthcoming). Applying this approach ultimately resulted in understanding the changes needed to better link elements and feedbacks to fit the goals of the system more tightly, and these are reported in Kansky (2022). We are not aware of other HWC studies that have specifically applied a systems thinking approach. We believe that understanding HWC and coexistence within this broader framing would have important policy benefits as it would allow for better multi-sector collaboration, coordination, and synergistic effects to improve management of the complexity of human–wildlife coexistence.

The *Nonviolent communication* element brought qualities of compassion, non-judgment, honest expression, trust, and equity to the learning process. We concluded this element was successful based on the large number of expressions of appreciation for the NVC part of the training (Table S5), the records of knowledge of the NVC concepts and ideas (Table S6), and the records of tangible attitudinal and behavioral changes that demonstrated increased compassion and empathy toward both people and wildlife (Tables S10, S11 and Kansky and Maassarani 2022). Evidence suggested these changes had persisted even after three years (Table S3). Issues of power, equity, and trust are seen as especially important in participatory processes but are not often addressed (Armitage et al. 2012; Suskevics et al. 2018); therefore, including NVC training in such processes could be especially beneficial. In addition, empathy has been found to be a driver of tolerance toward damage causing wildlife (Kansky and Kidd 2024) and framing animals as having universal needs proved effective in changing both attitudes and behavior toward wildlife. The role play with the elephant (week four table S2) was especially useful (see also Kansky and Maassarani 2022).

We taught NVC in the context of HWC, but it could easily be incorporated into other interventions to transform different natural resource conflicts. For example, our discussion of the good and bad things of living with wildlife to introduce the concept of feelings and universal needs (week one, Table S2) could be applied to a discussion on the pros and cons of a policy or management plan where there are disagreements between stakeholders. And our example of an incident where a person of authority did something in relation to wildlife that a participant did not like, which we used to explain the differentiation between observations versus interpretations (week three, Table S2), could also be applied to any difficult interaction between different stakeholders.

Although NVC has primarily been explored in education, care services, prisons, conflict transformation, and families (Till 2021), it is rarely applied in the environmental sector or more specifically in relation to human–wildlife conflicts. Recently, its potential has been highlighted by Williams et al. (2021), and our work supports its potential, especially in cases where the causes of conflict are deep seated and complex (Madden and McQuinn 2014; Zimmerman et al. 2020; IUCN 2023).

Finally, the *learning-based approach* proved useful to create a space for mutual learning and understanding by members of conservancies, conservancy management, and researchers. First, *transformative learning* focuses on individuals and how their ideas and understanding about the world transform based on rational discourse and critical reflection (Moyer and Sinclair 2020). Through this process, assumptions and beliefs are challenged and improved so they can be applied more effectively. It is therefore a theory of the link between learning and action. In our program, NVC was a useful tool to achieve transformational attitude and behavior change as measured by the stories participants shared in the feedback sessions at the start of each session when reflecting on how they applied the information from the previous week’s session (Table S1, Tables S2, S3, S10, S11, Kansky and Maassaraani 2022), as well as the evaluation after three years (Table S3). The NVC concepts made people rethink their ideas and values about compassion toward other people and wildlife and the experiential and relevant examples used to teach these concepts allowed participants to relate it more personally. The week in between each workshop gave them time to process, reflect and apply it in their daily lives. Transformation also happened at the empowerment level—participants reported changes in temperament from being more “ill tempered” to “softer” resulting in them feeling more empowered, content, and able to communicate and interact with more ease. Some theories about sustainability transformations emphasize the need for personal transformation for wider societal change (Moyer and Sinclair 2020) and NVC could be a useful approach to achieve this when incorporated into sustainability transformation processes.

Second, *social learning* focuses on the idea of individual learning by interacting with others in collaborative spaces and these individuals then going out to interact and share their new knowledge with others. In this way, the new knowledge reaches other groups, organizations, or institutions bringing about change in these spaces, ideally leading to improved management of social–ecological systems (Newig et al. 2019). The assumption is that the individual is the starting point, and in order for change to happen at other levels, the knowledge of one individual must be disseminated so that organizations or entire societies also change. In our program, we found evidence of knowledge transfer to the wider community as reported by participants during the program and after three years. Although this was not a specific goal of our program, we had hoped that by recruiting change leaders, encouraging them to formalize the groups at the end of the program and create learning opportunities with their communities, a transfer of knowledge and understanding would take place. From the evaluation after three years, it appears that participants were enthusiastic and showed initiative in spreading the knowledge leading to tangible outcomes suggesting that specifically focusing on social learning in program design could lead to even greater impacts.

Another aspect of social learning processes is deliberation—‘Social learning is the process of framing issues, analyzing alternatives, and debating choices in the context of inclusive public deliberation’ (Daniels and Walker 1996). Public deliberation, in turn, has been defined as a means by which ‘opinions can be revised, premises altered, and common interests discovered’ (Reich 1988). Deliberation implies ‘equality among the participants, the need to justify and argue for all types of (truth) claims, and an orientation toward mutual understanding and learning’ (Renn 2004). Although it was our intention to create the space for deliberation by inviting guests to join participants, in retrospect, our program was not able to achieve robust deliberation. Based on the questions participants asked the guests and the types of problems that emerged (Tables S2, S7, S9), it was evident that participants had little knowledge and understanding of how the system worked so most of the time was spent on knowledge acquisition with little time for debate and discussion. Thus, an important lesson is that deliberation can only happen after people have enough basic knowledge and understanding of a system and only after this can one develop opinions about the issues. This may be especially important where stakeholders have little access to information.

Third, *policy learning* focuses on acquiring new knowledge to inform policy by engaging state officials, policy networks, or policy communities (Cairney 2012, Suskevics et al. 2018). In this program, we intentionally did not engage policy stakeholders because these stakeholders are often the focus of consultations while local communities are underrepresented. Instead, through our program we hoped that participants would be empowered to engage with policy stakeholder and influence policy. This was achieved to some extent because participants reported attending more public meetings and sharing ideas from what they learnt in the workshops. Engaging with policy communities directly would be an obvious next step for future social learning programs.

## Limitations of the program

We concluded our program was generally successful and had a transformative impact for participants based on the high attendance rate, large number of records expressing appreciation, the large number of questions asked, reports of attitude and behavior change, and the reports of disseminating information from the training to others who did not attend (see also Kansky 2022; Kansky and Maasaraani 2022). We also concluded that participants trusted the space and felt safe based on some of the more difficult questions they posed to the invited guests from the management committee after learning about the policy documents. However, some limitations to the program were noticeable.

Firstly, the lack of adequate time for deliberation during the dialogues due to the general lack of knowledge about the policies and processes of how the conservancy operated. This, however, was unavoidable as we could not have anticipated the lack of information by participants. This highlights the importance and utility of a collaborative process of “unpacking the system” that includes both researchers and stakeholders as an important first step in engaging in transformative change.

Secondly, participants reported a lack of support from conservancy leaders when they tried to implement new projects or suggest policy changes. Conservancy members in leadership roles were not invited to participate in the program, and this may have contributed to their resistance to support participants as leaders felt the participants were now competing for their positions. Therefore, ideally it may be useful to include participants with leadership roles.

Thirdly, the NVC training focused more heavily on empathic connection with others (people and wildlife) than with self. At the time and due to time constraints, we thought this focus was more important, but upon reflection this was an omission as self-connection and self-care are equally important in growing NVC consciousness and personal transformation.

Fourth, inclusivity and power dynamics—as emerged through the discussions, a key issue in the conservancy is information flow—that members do not always get information about the conservancy. When we recruited participants to volunteer for the program, it is possible that many people did not get the information about the program or the date that we were coming to recruit people. This might be especially problematic for villages that are far away from the main hubs close to the main road going through the area. In terms of gender, age, and power dynamics, it would be unrealistic to think this did not play out in the workshops due to the still predominantly hierarchical and patriarchal traditional culture prevalent in rural Africa. However, by recruiting an equal number of male and females in each group, limiting the age of participants to below 45, and using Nonviolent communication, we feel we were able to mitigate against these factors to the best of our ability.

## Adaptation of program to other contexts

As calls increase for participatory and transdisciplinary approaches to deal with the complexity of human–wildlife conflict new tools and approaches are needed, and we hope our program can contribute to this emerging field. Programs need to be designed to the specific context. For example, in contexts where collaborative governance is already embedded in the social–ecological system such as CBNRM, learning programs such as the one we designed could be used to unpack the system to understand and identify where interventions are needed as well as build capacity for civic engagement (Kansky 2022; Kansky forthcoming). A second learning process could then be designed to modify, change, or redesign the system to improve resilience and adaptation (Kansky forthcoming). However, in social ecological systems where there is little collaboration in place and with a more diverse set of stakeholders, it would be necessary to first design a learning program to build trustful relationships and collaboration networks depending on contextual factors such as the level of conflict (Newig et al. 2019; Suskevics 2018; Zimmerman et al. 2020). Different learning modules could then be designed to address different needs, contexts and stages of the problem, with NVC training being a cross-cutting learning module to build trust, respect, empathy, and collaboration between people and tolerance toward wildlife. Continued NVC practice in these modules could further build NVC capacity in communities and within the system.

Embedding learning approaches into collaborative governance systems to ensure adaptation and resilience would be especially important for managing human–wildlife systems as both wildlife populations and stakeholder groups change over time. Bottom-up learning approaches with a focus on capacity building would also be important in the context of HWC because local people are most affected by wildlife but often have less power or capacity to promote their interests, especially in hierarchical cultural systems (Chanchani and Theivananthampillai 2009; Schwartz 2014).

## Conclusions

We applied a novel participatory learning-based approach to better understand why human–wildlife coexistence remains a challenge in Namibia’s communal conservancy programs. The program was based on three theoretical elements that have not yet explicitly been applied in the context of HWC, namely systems thinking, Nonviolent communication, and learning-based approaches. Based on our reflection on the program and its outcomes, we concluded the following:

(i) Training in NVC can result in multiple complementary benefits. Firstly, it provided the basis for convivial dialogue between stakeholders that were often in conflict, secondly, it enabled personal growth and empowered some participants who had been struggling with interpersonal communication within their families and communities, thirdly, it increased participants empathy and tolerance for wildlife which resulted in positive outcomes for wildlife from the implementation of mitigation measures to prevent damage and reduced hunting, and fourth, it enabled better collaboration between community members (for example in fire management and sharing information on how to behave when encountering wildlife) and between community members and conservancy management in general and specifically to promote human–wildlife coexistence (for example in doing outreach with the field officer on implementing mitigation measures).
(ii) Including a systems thinking perspective enabled both the researchers and participants to focus more broadly on understanding the whole social-ecological system rather than only focusing on technological fixes. With this approach, we were able to understand the shortcomings in the whole governance system and understand the complex interactions and processes between various stakeholders and the ecology of the landscape. Future interventions specifically applying a system thinking lens may go a long way in preventing the shortfalls from focusing on narrow technological fixes (e.g., Noga et al 2016).
(iii) Taking a learning perspective in dealing with HWC and promoting coexistence can promote transparency, build trust, increase capacity, promote equality and tolerance, and increase collaboration and mutual understanding of the system and issues. Programs such as the one we implemented can easily be adapted to other contexts using the foundations of the three elements, and as calls for environmental justice and real participation increase, we feel the program that we implemented can provide a useful blueprint.

## Supplementary Information

Below is the link to the electronic supplementary material.

Supplementary file1 (PDF 724 KB)
