# Supplementary material for: Participatory co-learning for human–wildlife coexistence: Reflections on a novel program applying systems thinking, nonviolent communication, and learning-based approaches
Source: Ambio. 2024 May 16;53(10):1479–91. doi: 10.1007/s13280-024-02032-5 (PMC11383895; doi:10.1007/s13280-024-02032-5)
Supplement: Supplementary file 1 — Supplementary file1 (PDF 724 KB) [file 13280_2024_2032_MOESM1_ESM.pdf]

Ambio

Electronic Supplementary Material This supplementary material has not been peer-reviewed.

Title: Participatory co-learning for human-wildlife coexistence – reflections on a novel program applying systems thinking, Nonviolent communication and learning based approaches.

Authors: RUTH KANSKY[1]*, TAREK MAASSARANI[2] AND JOERN FISCHER[3]

[1] Department of Conservation Ecology and Entomology, Stellenbosch University, Private Bag X1, Matieland 7602, South Africa.

[2] Georgetown University, Justice and Peace Studies, Washington, DC 20057

[3] Social-Ecological Systems Institute (SESI), Faculty of Sustainability, Leuphana Universitaet Lueneburg, Universitaetsallee 1, 21335 Lueneburg, Germany

Corresponding author: kanskyruth @gmail.com

Table S1. Session aims and outcomes (see also Table S2 for session content, key emerging issues and outcomes per conservancy). The number of participants attending each week with the percentage out of a total of 54 participants is also indicated.

<table>
<tr><td>

Week one: Introduction to nonviolent communication through reflecting on benefits and costs of living with wildlife
No. attending:53 (98%)
Aims:
1. Understanding needs met and unmet in relation to living with wildlife for researchers to understand context and for participants to understand each other’s perspectives, be heard and receive empathy.
2. Introduction to key nonviolent communication concepts - feelings and universal needs

</td></tr>
<tr><td>

Social Learning outcomes:
• enjoyed learning from others in the discussion and hearing others wildlife stories
• felt hopeful that the understanding and knowledge will be useful
• shared lessons with family and others
• shared story of a zebra that came near to village – discussion of good things about wildlife made them appreciate watching the zebra and used the opportunity to tell children about them
Nonviolent communication outcomes:
• appreciated fact that animals can also have universal needs

</td></tr>
</table>

- appreciated hearing story of rescuing a buffalo- empathy and care towards wildlife was a new idea and saving wildlife contributes to increasing wildlife numbers. Normally if they found an animal in distress it would be killed for the meat
- understood universal needs and feelings and how it could help to reduce conflict and find better solutions
- appreciated importance of listening and respecting one another even if you have different opinions, for example, that people can have different views about wildlife
- understood that people have different feeling and needs which can lead to different ways of doing things

System outcomes

- new knowledge that meat in Zambezi cannot be exported due to interactions of cattle with wildlife
- appreciated that positive and negative aspects of living with wildlife must be well managed to ensure coexistence and human wellbeing
- learnt from story of a hippo attack that they attack when protecting young
- appreciated understanding positive and negative things about living with wildlife
- learned about how to behave when encountering wildlife and reported numerous examples of applying the new knowledge (see Table S1 for examples)

Week two: Building nonviolent communication knowledge and practice through future visioning
No. attending:51(94%)
Aims:

1. A self-exploration and sharing by participants of what life is like in the present living in conservancies with wildlife.
2. A self-exploration and sharing of participants' visions for the future in the conservancy that would improve human-wildlife coexistence.
3. To learn about a key differentiation in nonviolent communication – *universal needs* versus *strategies*

Social learning outcomes

- appreciated contributions from others in the workshop
- appreciated the group work and getting different perspectives on their conservancy- the positive and negative aspects
- discussed with elders in community about the past and future of the conservancy

Nonviolent communication outcomes

- understood difference between universal needs and strategies – for example, that cars, cell phones and money are not universal needs. This brought ease and less stress as well as appreciation for the non-monetary benefits of life, like reciprocity
- understood that both wildlife and people have needs that need to be cared for
- understood that coexistence with wildlife was possible – seeing zebras by the village and the dialogues in the workshop brought awareness that there are different ways to coexist with wildlife and there are positive ways to interact and teach children about wildlife
- started appreciating that identifying universal needs can help reduce/prevent conflict
- showed concern about how others not attending the program could be informed to

make decisions about conservancy

- realized that good communication and respect are ingredients for good relationships between the community and conservancy management
- understood the difference between universal needs and strategies and that there is no conflict between needs but there may be between strategies to meet needs
- appreciated the universal needs of cooperation and love
- appreciated lessons in general as learnt about ways to avoid conflict and enhance coexistence between people and people and animals
- participants reported examples of how they applied nonviolent communication skills and empathy (see Table S1)

System outcomes

- improved understanding of the conservancy and how things might change - felt hopeful
- understood importance of implementing mitigation measures to prevent damage
- understood that living with wildlife has costs and benefits
- appreciated that wildlife and people should be separate, and each should have their own area
- understood that conservancy finances should be well managed and that the budget should be shared with community members
- understood importance of not cutting down trees as there would be no food for animals
- realized the need for people with good communication and empathy skills to deal with people who incur damage from wildlife and that this would promote coexistence
- realized that the community has power and should elect leaders with relevant skills
- realized that mismanagement by leaders is “bringing the conservancy down”
- realized that the conservancy does not generate enough benefits so there is a need to diversify income
- appreciated reflecting on the good and bad of living with wildlife and learning from each other how to conduct themselves when encountering animals
- many applied the ideas of how to behave when encountering animals in the bush (see examples Table S1)
- learned about the damage compensation process and which species could be claim for
- understood that building in the wildlife corridors is not good.
- understood that animals mostly attack people when they are provoked

Week three: Nonviolent communication training – observations versus interpretations through unpacking conservancy conflicts
No. attending:47 (87%)
Aims:

1. To learn a key nonviolent communication concept – *observations* and a key differentiation between observations and interpretations
2. To practice this differentiation using examples of conflicts between community members and conservancy management.

Social learning outcomes

- shared nonviolent communication principles of empathy and tolerance with others
- appreciated learning in the group and sharing stories

- had discussions with elders after the workshop around good and bad of living with wildlife and realized it would be better to have wildlife with better leadership

Nonviolent communication outcomes

- appreciated the concept of empathy – that it brings tolerance and if you listen first, you are more likely to get your needs met
- reported many examples of showing empathy towards wildlife and finding different strategies to live with wildlife (see Table S1)
- reported examples of self-improvement by being more tolerant and less ill tempered
- appreciated and became aware of specific universal needs such as love and empathy in the group and how respect and cooperation can avoid conflict
- noticed the difference between *observations* and interpretations and that when you see people making interpretations that are not based on observations it can cause pain and conflict
- understood the importance of listening and not interrupting and that when someone says something bad one should not get angry but try to understand where the person is coming from (their universal needs)
- reported examples of how they used nonviolent communication for better connection, compassion, and conflict avoidance (see Table S1)

System outcomes

- the example of a negative interaction with manager during led to an understanding of the necessary leadership skills, including showing respect in interactions with the community
- understood the problems the conservancy was having with the professional hunter - that he did not pay up or implement the development projects agreed on
- applied the concept of *observation* versus interpretation and looking at the wind direction to avoid an elephant encounter (see Table S1)

Week four: Nonviolent communication training – empathy and making requests through a role-play conversation with an elephant

No. attending:43 (80%)

Aims:

1. To learn about cultural stories of wildlife species to understand how or if they impact human tolerance towards different species
2. To learn two key nonviolent communication concept – requests, empathy, and empathic listening
3. To learn about a key differentiation in nonviolent communication – requests versus demands
4. To practice empathic listening, using an elephant in a question-and-answer role play, for participants to understand that animals also have feelings and universal needs

S**ocial learning outcomes**

- learnt new things from the cultural stories

Nonviolent communication outcomes

- became aware of empathic concern in general - before workshops the participant would not think to “bother about friends’ problems

- reported examples of empathic behaviour change towards wildlife and people (see Table S1)
- realized that coexistence with wildlife is possible
- understood the difference between request and demand - how to best approach someone and when hearing a "no" one can try to find other requests and not give up
- elephant role-play - learnt to see things from an elephant perspective, realized that animals are like people in that they have similar feelings and needs, that elephants would also like freedom to roam freely without being chased by people, that humans are not empathic to elephants needs by building in corridors
- understood difference between empathy and sympathy and that when one responds empathically to someone the other person responds nicely

System outcomes

- role-play with elephant was impactful - brought realization that need to keep corridors open (elders told us that but we did not listen) and apply mitigation measure because elephants are just trying to feed themselves
- expressed hope that relations with conservancy management could improve

Week five: Understanding conservancy policy documents and unpacking conservancy governance issues from dialogues with guests
No. attending:45 (83%)
Aims:

1. To learn about the policies that govern conservancies
2. To get skills and practice presenting to an audience
3. To ask questions and have discussions with an invited guest to learn and understand what is working and what are the challenges in the conservancy
4. To practice nonviolent communication skills in dialogues with the guest

Social learning outcomes

- felt hopeful that things could be improved in the conservancy with new understandings
- appreciated the guest's honesty and thought he knew his job well
- community members not attending the program expressed interest in the workshops and a participant shared what they learnt about human-wildlife conflict and damage reporting

Nonviolent communication outcomes

- appreciated that the dialogue went well with the guest and that there was mutual respect between him and the group
- realized the importance of the universal needs of cooperation and love - that game guards need to cooperate among themselves to help farmers, that neighbors need to love each other to provide reliable evidence when making damage claims, and that love is needed for people to have empathy and do their job with passion
- showed empathy towards game guards - that maybe they are busy sometimes and can't always attend to them immediately
- realized conflict results when universal needs are not met for both people and animals

- thought everyone should get nonviolent communication training if we want people to help each other.
- a participant wanted to know how sick animals could be helped

System outcomes

- understood why conservancy is not going well - because the constitution is not being followed
- appreciated understanding how damage reporting and compensation works, which species are compensated (some thought all animals were compensated), new amounts according to new policy, should measure fields as the claim is based on the size of fields, that maybe they do not prepare themselves enough to make damage claims and that's why game guards sometimes reject their claims
- understood reasons there is not enough money to pay compensation for all species because there are no campsites or lodges to increase income. Felt hopeful this could change
- understood that some game guards are not skilled enough to do their jobs and that may be the reason the conservancy is not working well
- felt compensation amounts in the national human-wildlife conflict policy are not in proportion to the importance of crops for peoples' livelihoods
- understood that conservancy management does not follow the constitution in relation to payments –they get monthly salaries while constitution says they should get paid per meeting attended
- appreciated the new knowledge from the policy documents and guest
- learnt about some new mitigation measures for elephants
- heard that game guards are supposed to conduct awareness about the use of mitigation measures, but the participant has never seen that happen
- felt that people writing the policy documents do not consult the community as they are not relevant to local conditions
- knowledge from the constitution was useful as a participant could explain the rules during a meat distribution event at their conservancy based on his new knowledge of the constitution
- appreciated the guests' explanations about the problems and complexities with dealing with compensation claims
- learnt new information about early burning, that small animals die but big animals gain from the new grazing.

Week six: Understanding conservancy policy documents and unpacking conservancy governance issues from dialogues with guests
No. attending:42 (78%)
Aims:

1. To learn about the policies that govern conservancies
2. To get skills and practice presenting to an audience
3. To ask questions and have discussions with an invited guest to learn and understand what is working and what are the challenges in the conservancy
4. To practice nonviolent communication skills in dialogues with the guest

Social learning outcomes

- one group were generally disappointed with their guests
- guest expressed gratitude for workshops
- a different group expressed appreciation for their guest, in his knowledge about policies and how well he explained things
- felt inspiration from the guests' motivation to get involved in conservation and his knowledge in answering questions
- all information was very useful

Nonviolent communication outcomes

- reported examples of applying empathy towards people and wildlife (see Table S1)
- conflict in one group emerged during checkout about interruptions during the dialogue with the guest and we resolved it using nonviolent communication in the group
- a participant applied the concepts of *observation* versus interpretation to ask the guest a question

System outcomes

- appreciated learning about how to behave when encountering elephants in the bush
- appreciated learning about different mitigation measures to prevent damage
- found the conservancy human wildlife conflict policy useful and to learn that since 2015 compensation had not been paid
- understood the importance of measuring field size to make a damage claim
- learnt how area representatives communicate with members about meetings
- increased trust between community and area representative - after the discussion about the lack of communication by area representatives during the workshop, the following week the area representative SMSed participants about a meeting which was well attended
- expressed surprise learning that compensation for injury or death will only be given if incident occurred in or near the village as most of the danger is by the river
- knowledge about the constitution brough clarity on how the conservancy works
- knowledge about electricity contractor helped not blame him- she thought he just ran out of money
- expressed disappointment around answers about progress on development project and delays caused by Ministry of Environment Forestry and Tourism for lodge approval but others felt hopeful that lodge problem would be resolved soon, the electricity project will get finished and the conservancy will get more income
- some felt discouraged at inefficient use of funds, unfinished projects, and to learn the hunter is not paying
- learned that Ministry of Environment Forestry and Tourism was responsible for quota setting for wildlife hunting

Week seven: Empathic listening practice and unpacking conservancy governance issues from dialogues with guests
No. attending:38 (70%)
Aims:

1. To practice empathic listening by guessing feelings and needs

2. To ask questions and have discussions with an invited guest to learn and understand what is working and what are the challenges in the conservancy
3. To practice nonviolent communication skills in dialogues with the guest

Social learning outcomes
- some felt the guest was avoidant in answering questions
- facilitator felt discussion did not go well as participants were not using communication skills in the dialogues
- some felt hopeful that things can change once they learn and understand better about the conservancy through the workshops and if new leaders come in things could change
- group expressed appreciation for the information

Nonviolent communication outcomes
- group gave the guest empathy when she expressed that she felt sad when they laughed at her for not being able to respond to questions. She was happy with the empathy given.
- Reported examples of empathic behavioral change towards humans (see Table S1)

System outcomes
- felt management were in their positions too long, with yearly promises of changes, but the same problems remained.
- found information useful that conservancy were creating cutlines to count wildlife
- reflected on knowledge they learnt about wildlife diseases showing good knowledge retention after one week
- appreciated the knowledge from the guest about wildlife diseases and the issue of expense of quarantine for the sale of meat outside the Zambezi region.
- appreciated suggestion by guest that community should get trained to be professional hunters themselves rather that outside people
- there was meat distribution during the week and a participant expressed uncertainty about the health of the meat

Week eight: To practice using nonviolent communication concepts and unpack conservancy governance issues during dialogues with guests
No. attending:38 (70%)
Aims:
1. To practice using nonviolent communication concepts
2. To ask questions and have discussions with an invited guest to learn and understand what is working and what are the challenges in the conservancy
3. To practice nonviolent communication skills in dialogues with the guest

Social learning outcomes
- appreciated skills, knowledge, openness, and honesty of guest
- guest expressed gratitude to group and felt their interest showed love for the conservancy

- guest expressed gratitude to the research team and for the format of the program as a tool for understanding and dialogue – he expressed he had been apprehensive about the research at first
- guest made suggestions on how the group could be involved in the future of the conservancy

Nonviolent communication outcomes

None

System outcomes

- understood what needs to be done to improve conservancy and felt inspired to move forward on that
- new knowledge increased trust for conservancy management
- realized problem of lack of communication by conservancy management to community and control of knowledge by indunas so not everyone is heard
- appreciated learning about the delays in getting the lodges and that the chairman is elected at the annual general meeting

Week nine: World café on group projects, nonviolent communication assessment, final reflections and program certificates

No. attending:49(91%)

Aims:

1. To evaluate participants knowledge of nonviolent communication
2. To discuss project ideas for the groups to implement post learning program
3. To celebrate end of program with presentation of certificates of participation

Final checkout

Social Learning outcomes

- expressed hope that the group will pass on knowledge to the community to resolve conflicts and be active in solving problems in conservancy
- enjoyed learning from each other and appreciated the improved sharing of food in the group – at the beginning everyone took first without considering others
- general appreciation for workshops – received hook and not just fish, feel privileged to have received knowledge and skills
- thanked conservancy management for allowing the program to take place

Nonviolent communication outcomes

- appreciated the nonviolent communication training - that animals also have universal needs and that understanding increases respect for animals
- empathy and understanding towards wildlife - changed a participants' behaviour to stop hunting opportunistically with his dogs, empathy enabled a participant to think of conserving rather than destroying wildlife, to have compassion for small animals that get destroyed during fires, feel general love towards animals, and understand impacts of humans on wildlife.
- understood that coexistence is possible
- appreciated the nonviolent communication skills which have improved communication, reduced conflicts between people and changed personality to be more

agreeable – shared stories of mediating conflicts, treating children with more compassion and not beating them, respecting and being open to different viewpoints, better relationships and tolerance with siblings and other community members, changing cultural values (seeing wife as more equal) and feeling general love towards people (See Table S1 and Kansky & Maassarani 2022 for narratives)<br>• appreciated the difference between universal needs versus strategies – that money and cars are strategies<br>• used nonviolent communication skills and concepts – used observation to understand why cattle are coming into his yard, then used improved communication skills to resolve conflict with neighbor over cattle, making requests rather than demands and able to find solutions to meet everyone's needs<br>**System outcomes**<br>• program helped to improve coexistence with wildlife<br>• program helped improve the relationship and understanding with conservancy management<br>• better understanding of the importance of zonation plans and wildlife areas for campsites and lodges to increase income for conservancy so there are more funds for compensation of damage<br>• better understanding of conservancy – more appreciation but better leadership and skills are needed<br>• better understanding of Ministry of Environment Forestry and Tourism

Table S2. Human-wildlife coexistence learning program. A description of the aims of the program, lesson plans and content, key nonviolent communication concepts and ideas, summary of emerging issues and outcome of each week from participant feedback at the end of each session and after one week in between weekly sessions. CMC=conservancy management committee, HWC=human wildlife conflict, NVC=nonviolent communication, MEFT=ministry of forestry and tourism, universal needs=universal human needs, PH=professional hunter

**Week one: Introduction to nonviolent communication through reflecting on benefits and costs of living with wildlife**<br>**Aims:**<br>**3.** Understanding needs met and unmet in relation to living with wildlife for researchers to understand context and for participants to understand each other's perspectives, be heard and receive empathy.<br>**4.** Introduction to key nonviolent communication concepts - feelings and universal needs
**Lesson content:**<br>• Introducing feelings and needs through discussion based on two questions:<br>- What are the things that you enjoy about living in an area where there's lots of wildlife?<br>- What are the things that you do not enjoy about living in an area where there is lots of wildlife?

- During the discussion one facilitator writes on flipchart a list of feelings in one column and a list of needs in a second column. One page is dedicated to positive feelings and needs satisfied while a second page is for negative feelings and needs unsatisfied.
- After discussion presentation of concepts of feelings and universal needs

Nonviolent communication concepts and ideas for the lesson:
Universal needs:
Universal needs are:

- Shared by all humans and some animals
- Positive
- Not specific to a place, person, time or object
- Examples: respect, connection, understanding, to be heard, autonomy, freedom
- universal needs are different to more specific economic “wants” and “desires” for e.g. I want a car, I want a chocolate. These are strategies to meet needs, for example a car could be a strategy to meet the universal needs for safety when walking in an elephant corridor, or it could meet a universal need for respect if that is a social norm in a community.
- Feelings are the inner expression of our universal needs. When universal needs are being satisfied, one experiences positive/desirable feelings. When needs are not being satisfied, we experience negative/undesirable feelings. As long as we are alive, we are always experiencing feelings, regardless of whether we are aware of them or not. Our feelings are dynamic, often changing every few seconds. By training ourselves to be more mindful of our feelings, we gain a greater understanding of and connection with our needs.

Summary of emerging issues: Group 1

- many expressions of positive aspects of wildlife: learning about animals, meat, beauty, income from trophy hunting, jobs through tourism, hunting and conservancy, tourism allows meeting with international people which gives a sense of a global village, appreciation of interconnectedness of nature, passing knowledge to youth, opportunities to socialize during meat distribution, tractor, sense of pride knowing animals belong to conservancy, connection to grandfather through animal stories, ability to support family through provision of meat, enjoyment seeing animal behavior, dancing wildebeest, positive experience of rescuing a buffalo stuck in mud.

- negative aspects of living with wildlife: crop and livestock loss, compensation does not cover full costs, meat from Zambezi cannot be sold outside the province due to wildlife disease control measures and lowers value of cattle, since locals survive from farming they bare most of costs of living with wildlife while poachers and tourists benefit, death and injury from wildlife, psychological costs for e.g females feel particularly vulnerable, sadness and hopelessness when one loses all ones hard work,

- Other problems highlighted were lack of support and understanding from authorities and conservancy management of what it’s like to live with wildlife, damage by small species are not compensated for, unequitable benefit distribution, complaints of community are not reaching MEFT, game guards don’t have weapons to scare animals

from fields, not enough meat to go around to make a difference to food security,

Outcomes

End of lesson

- appreciation of fact that animals can also have universal needs
- new knowledge that meat in Zambezi cannot be exported
- enjoyed learning from others
- feeling hopeful that understanding and knowledge will be useful
- appreciation that positive and negative aspects of living with wildlife must be well managed to ensure coexistence
- enjoyed learning from others is the discussion and hearing others wildlife stories
- appreciated hearing story of rescuing buffalo - empathy and care towards wildlife is a new idea and that saving wildlife contributes to increasing wildlife numbers. Normally if an animal is found in distress it would be killed for meat
- understanding of universal needs and feelings and how it can help to reduce conflict

After one week

- shared lessons with family and others
- appreciated that wildlife must be well managed to ensure wellbeing of people
- enjoyed learning from others
- appreciated story of rescue of buffalo by one participant-felt touched by care shown to wildlife
- shared story of zebra that came near to village –discussion of good things about wildlife made them appreciate watching the zebra and used opportunity to tell children about them
- understanding and awareness of feelings and needs
- appreciating that people have different views about wildlife

Summary of emerging issues: Group 2

- many expressions of positive aspects of wildlife: meat, forex, jobs, water tanks, electricity, end of year cash, money from selling crafts, funeral cover, enjoying watching animals, existence value
- negative aspects of living with wildlife: women constantly fear of elephants when going to river to collect reeds, crop damage, livestock damage, damage to canoes
- Many stories of encounters with wildlife-negative experiences of being chased or attacked by animals: attack by hippo while fishing and canoe broken, a community member was killed by a hippo, a participant was chased and attacked by a buffalo – he has scars, a woman was chased by elephant while working in the bush-she now fears elephants even when she sees footprints, a hippo attacked a tourist boat who fell into the river and lost his camera, a friend was chased by hippo when collecting reeds
- eight out of 18 in group had been attacked by animals

Outcomes

End of lesson

- appreciated understanding positive and negative things about living with wildlife
- learned about how to behave when encountering wildlife, e.g looking at wind direction
- appreciated the communication skills - to listen and respect one another even if you have different opinions
- appreciated universal needs of connection - how to be friends with one another

After one week

- many new stories about negative experiences with wildlife but with a different perspective after the lesson; a participants fathers cow got killed by a lion, while he investigated, he came upon the lion but did not run away-something he had learnt from the discussion in week 1, a participant told a story of a women who walked into a lion at the river while collecting reeds but was wise old woman who told the others to back off slowly so the lion did not attack them-this consolidated the learning from the week 1, a participants aunt came across wild dogs while coming back from river and managed to fend them off with her sickle but then wanted to go and get a gun to kill them. He tried to convince her not to do that explaining to her that they should try to coexist with them and ensure future generations will see them, a participant shared about not lighting fire to get elephants to change direction, a participant came across a heard of elephants while herding cattle but walked around them so as not to disturb them.

Summary of emerging issues: Group 3

- many expressions of positive aspects of wildlife: meat, wealth to nation, small cash income, tusks, skin from animals to make things, foreign exchange for country, learning about animals and passing on knowledge to youth, experiential knowledge about wildlife
- negative aspects of living with wildlife: crop loss, negative emotions from wildlife impacts when you see your entire harvest being eaten by an elephant, a crocodile eating your prize ox, feeling hopeless when you try to chase wildlife, but they don't go
- many negative experiences with wildlife: being attacked by a crocodile and thinking your life is about to end, being attacked in a boat by a hippo and having to jump out and hide in reeds
- other issues: compensation does not cover costs, there are delays in compensation payment
- expressed desire to live with wildlife but need to find solutions to the high costs

Outcomes
End of lesson

- understanding that people have different feelings and needs which leads to different ways of doing things
- understood idea of considering others, e.g if someone is busy and can't help at a specific time to come back another time
- learnt from story of hippo attack that they attack when protecting young

After one week

- appreciated understanding positive and negative things about living with wildlife

- feelings and needs are useful to find better solutions
- ideas of feelings and needs were shared with others not in group
- appreciation of universal need “support” to enhance empathy towards others, e.g sharing resources with those who have little. E.g shoes

Week two: Building nonviolent communication knowledge and practice through future visioning

Aims:

4. A self-exploration and sharing by participants of what life is like living in conservancies with wildlife.
5. A self-exploration and sharing of participants’ visions for the future in the conservancy that would improve human-wildlife coexistence.
6. To learn about a key differentiation in NVC – *universal needs* versus *strategies*

Lesson content:

- Sculpture building based on U theory[1] and two scenarios:
  - Build a sculpture of a scene that shows your life right now where you are living in a conservancy with wildlife. Include your relationship with the conservancy office.
  - Change or modify the scene/your sculpture to show how you would like to see your life in the future. Think of the changes you would like to see in relation to living with wildlife and the conservancy.
- Participants were divided into groups of 3-4 and given various items to build their sculptures such as pipe cleaners, Prestik, play dough, seeds and sticks[3]
- After each sculpture each group described their sculpture to the other groups
- Next, facilitator elicits strategies that came up in the future sculptures of the change’s participants want to see. Facilitator chooses two strategies that may conflict with each other. Identify universal needs behind each strategy. Demonstrate that strategies can be in conflict but the universal needs behind them are not.
- Have a discussion on what information and skills one would need to implement some of the strategies.
- Homework: Notice when you get into a conflict or disagreement with someone and when you're in disagreement or conflict with that person, try to imagine what are the universal human needs that person is trying to satisfy with the strategy that you are in conflict with. Then see if you feel different about the person or the conflict. If you have a journal, write about it.

NVC concepts and ideas for the lesson:
Universal need versus Strategies:

1. Strategies (actions or behaviors) by people or animals are attempts to satisfy universal needs. We can use many different strategies or actions to satisfy a need.
2. Disagreement and conflict occur when different strategies are incompatible with each other. Universal needs are never in conflict because, by definition they are universally positive. Therefore, they are a source of understanding, connection, compassion and creative problem solving.

3. For example, fencing in wildlife to create a park where visitors come to see them versus no fencing - Needs for fencing are freedom and choice for humans to move around; Needs for no fence are freedom for wildlife to move around freely in landscape. So, we disagree on strategy, but universal needs are same. In that way we can appreciate needs are not in conflict.

Summary of emerging issues: Conservancy 1

Sculpture stories

- Group one: Present - depict a farmer who incurred damage from wildlife to his crops and went over to the conservancy office to complain. But after speaking to the conservancy, he came out feeling hopeless that nothing will probably be solved, and he doesn't know what to do because he wants to live with wildlife, but he doesn't know how to without incurring costs. Future: There is now electricity in all the villages, not just at the conservancy office, there has been a good harvest now, the animals are in the park fenced in and they have water there and there are tourists who come to see our wildlife, there are projects that are being done and there is some wildlife in our conservancy, and we have brought our children to see them. There are now good relations between the conservancy and the community.
- Group 2: Present - describe a scene where a game ranger on patrol found a snared buffalo. He called a meeting in the village and explained that they don't like to see animals snared but the community should rather go to the conservancy office and ask for a local hunting permit. Then the game guard went on another patrol and found the buffalo near the river and reported to the community to say they are nearby, and people should not put our snares. Future: things have improved and there is more harmony with the people we work with in the conservancy. There is now a fence around the village which keeps wildlife away. There is a man standing in his field with his gun but now the gun is dropped because he is getting compensation for the damage. There are two men standing over there and the one is explaining to him the benefits of joining the conservancy – that there is electricity, a house, compensation and wild animals don't cause damage.
- Group 3: Present - describe the costs of living with wildlife - a predator proof kraal and a farmer who has a problem with a hyena. Since there is no help from the conservancy the farmer has a gun to scare away the hyena. There is also a field with an elephant and porcupine eating. The farmer is sad and angry because even if he reports the porcupine, he won't get compensated for that, so he shoots the porcupine. Future: there is a fence around the park and the wildlife are in the fenced area and they have enough water and food there, so they don't need to go and eat people's food and attack the cattle. There is a cattle kraal and an area for grazing, so they don't go in the park to graze. There are some tusks there which we have been given and we have sold them and bought some cars, and you can see me driving here is my car and I am at ease now. There is also electricity.
- Group 4: Present - describes a field being destroyed by elephant and porcupine. The farmer calls the game guards to help but does not get any support and he is very angry because he never receives any compensation. Future: There is a power line representing electricity, there is a corridor for wildlife to pass through from Botswana,

there is a windmill where animals can get water from, wildlife numbers have increased, there is a tree that is bent over representing the damage caused by wildlife, there is a person who is feeling happy that he has got some compensation now and he has electricity and water for cattle so he is happy that everything is now the way he wants.

- Group 5: Present - describes the conservancy with a big tree that represents a source of food and shelter for all the animals including birds and ants. In another area are the fields and they are dry because of the drought. There is an elephant with a man standing next to it and it represents how we preserve our wildlife. So overall the sculpture represents how everything depends on everything else to survive. Future: there are now power lines from the electricity project, there is a new tree in the center of the conservancy that represents a new community project so that they can get some relief from the drought. There are also cars and a man next to his field with his arms raised up. This represents the community and shows that they are being helped slowly so that the good outweighs the bad. There is also a book which represents a meeting where the community is gathering to decide on some change in rules, so they are better compensated.

Outcomes:
End of lesson

- improved understanding of conservancy and how things might change - feeling hopeful

After one week:

- appreciated that money is a strategy and not a universal need - brings ease and appreciation for non-monetary benefits of life like reciprocity.
- appreciated contributions from others in the workshop
- understood importance of implementing mitigation measures to prevent damage
- understandood that wildlife has costs and benefits
- understood that wildlife and people each have needs that need to be cared for
- appreciated that wildlife and people should be separate, and each should have their own area
- understanding that conservancy finances should be well managed and should share the budget with community members
- understood importance of not cutting down trees as there would be no food for animals
- that coexistence with wildlife is possible – seeing zebras by the village plus the dialogues in workshop made him more aware that there are different ways to coexist with wildlife and there are positive ways to interact and teach children about wildlife
- realized the need for people with good communication and empathy to deal with people who incur damage from wildlife and that this will promote coexistence

Summary of emerging issues: Conservancy 2

Sculptures
Group 1: describe the village with the conservancy office and some wildlife
Group 2: describe a village with the conservancy, some wildlife, a wildlife corridor with elephants going through to the river and a kraal with cattle.
Group 3: describe a village with some animals and trees

Future:
Group A: We had a community meeting to decide how best we can live with wildlife, and they decided to build a fence to keep the wildlife out of the village. Then they made an entrance gate and charged tourists to come in. There is a windmill inside the park for water, so the animals don't come out to the river by the village. We have our field here and we are harvesting many watermelons.
Group B: They describe the conservancy where animals are living side by side because wildlife are now used to people, and they are not troubling each other. For the cattle there is a cattle herder that keeps the cattle away from the animals but if some cattle are taken then they get compensation.
Group c: There is an electric fence that is around the village to protect people from wildlife, there is electricity in all the houses, there is a lodge and they come to the conservancy and hear about how people are living with wildlife, there are separate water areas for people and wildlife.
Group D: There is a separate park for wildlife and there is a hunter that comes to hunt wildlife and the money comes to the conservancy
Group E: We are still living in the traditional village, and we are still living with wildlife, and we protect them because they bring good things to us. For example, there is electricity and water. There is a farming project with different crops and there is a fish farming project where tourists can come and fish as well. There are people who do traditional dancing for tourists to come and see, there is a craft market

Outcomes:
End of lesson

- understood the difference between needs and strategies and there is no conflict between needs but there may be between strategies to meet needs

After one week

- appreciated reflecting on good and bad of living with wildlife and learning from each other how to conduct themselves when encountering animals
- appreciated that money is not a universal need and felt more at ease and less worried about not having money or feeling inferior
- appreciation of universal needs of cooperation and love
- appreciation of lessons in general as ways to avoid conflict and enhance coexistence between people and people and animals
- some applied ideas of how to behave when encountering animals in bush: watching impala from close and not disturbing them by using concept of wind direction, not disturbing hippopotamus foraging next to river-before would have run away or made a fire, a female participant who had in the past had a very scary incident with elephants, is less scared now that she knows how to skirt it using wind direction,
- some applied NVC skills and empathy- a male participant identified his wife's' need for support and empathy to stay with him at the hospital. In the past he would have just left her there because he had things to do.
- Appreciated universal needs of cooperation (after a negative incident with his friend and his dog who chased an animal, got bitten by a snake and died), love (if everyone applied it they would not be burning grass and making animals suffer)

- Identifying universal needs and applying them to show empathy- example of showing empathy towards a friend who lost a game they played together.
- Learning about compensation process and which species one can claim for
- Appreciating lessons as they are showing better ways to avoid conflict
- Understanding that building in corridors is not a good idea.
- Understanding that animals mostly attack people when they are provoked

Summary of emerging issues: Conservancy 3
Sculpture stories
Present:
Group 1: Give a general description of the animals and natural resources in the conservancy and the things they are used for. There is also a predator proof kraal.
Group 2: Present - describe small animals in the conservancy, a camelthorn tree that provides food for many animals including cattle and elephants, and when it eats the seeds, it disperses them. The porcupine and bushpigs are a problem but the conservancy does not pay and they don't understand why. Future: The conservancy is still there but there is a tourist lodge which brings more employment. There is also still hunting which brings income. But a park has been created and water has been diverted from the Zambezi River to the Kwando and it acts as a buffer zone between the park and the village.
Group 3: Describes village made of traditional/natural material where animals can pass through freely. They have fences made from natural materials, so these natural materials make the animals feel comfortable because they are not strange and scary materials. There are many buffalo in our conservancy but although animals sometimes destroy our fields, we have found a way to live with them.
Group 4: Describe a typical village and some of the natural materials that are used

Future:
Group A: Village has changed to a town with streetlights, roads, and houses made from bricks with a wall, water and electricity. There are cars in the town. The conservancy is still there but it has made a park where the animals can stay in harmony as we are staying in harmony on our side in the town. We no longer need to fetch water from the river as we have taps in our houses, we don't have to get food from the bush as we can just buy in the shops. So now we all live in harmony as we just go to the parks when we want to see animals. We all have jobs, and everyone is thriving, and we are living in harmony with the wildlife
Group C: The conservancy has been given as extra 20km from the neighboring national park because currently the conservancy is too small and there are not enough animals in it for hunting. A tourist lodge has been built. The river is the boundary, and the park is on the other side. On the other side is the village. But we don't feel very hopeful about the future of the conservancy
Group D: They don't think the conservancy will exist in the future because people are building in the corridors and already now there are not enough adult elephants to hunt because the conservancy is too small. There is not enough space for people and wildlife. We would like the conservancy to exist in the future with all the benefits that were predicted when it started in 1999- electricity, water.

Outcomes:

End of lesson

- start appreciating that identifying needs can help reduce/prevent conflict
- appreciated the group work and getting different perspectives on their conservancy- the positive and negative aspects
- showing concern on how others not in SL group will get the information and be informed to make decisions about conservancy. E.g on whether it should close or not
- new realization that community has power to elect leaders with proper skills

After one week

- understanding difference between universal needs and strategies - that cars, cell phones and money are not universal needs and that brings ease and less stress
- noticing developments in the area are unfolding as she predicted in the future sculptures
- realization that it is mismanagement by leaders that is bringing the conservancy down
- realization that good communication and respect are ingredients for good relationships between the community and the conservancy management
- spoke with elders and asked how conservancy in past was - they said it was better managed, so then he realized no need to close conservancy, just elect better leaders
- discussion with elders - they thought conservancy should not be dissolved as it acts as the umbrella for all the villages
- realization that the conservancy does not generate enough benefits so there is a need to diversify income

Strategies suggested from all sculptures:
Build strong wire fences around fields; Employ more game guards to guard fields; Stop professional hunters from hunting (as they make animals aggressive); Selecting new conservancy leadership who will distribute benefits equally/know how to manage conservancy/represent different families and views; Increase prices of trophy animals and pay in USD; Humans live closer together in villages separate from animals; Dissolve the conservancy; Conservancy gets an extra 20 km from Babwata National Park to increase land for wildlife for conservancy to use; Build stronger fences; Bring more tourism; Divert channel from Zambezi to Kwando; More benefits from wildlife; Avoid deforestation; Fence wildlife into one area; Communities buy and sell protected species; Put electricity for conservancy members; Separate water sources for domestic animals; Put fire breakers; Create wildlife corridors; Have windmill water pumps for wildlife; Educate communities about wildlife; Better compensation for damage; Create species database that is accessible to community; Separate wildlife from humans with a fence; Organize awareness workshops; Go around wildlife if you know its there; Live closer to wildlife so they get used to people; Install wind-powered water pump for wildlife; Connect village to electricity; Getting tourists to pay for watching wildlife; Build electric fences; Use lights to deter wildlife

Week three: Nonviolent communication training – observations versus interpretations through unpacking conservancy conflicts

Aim:

1. To learn a key NVC concept - *Observations*
2. To learn about a key differentiation in NVC – observations versus interpretations
3. To practice this differentiation using examples of conflicts between community members and conservancy management.

Lesson content:

- Revision of *Universal Needs* definition and homework
- Introduce concept of observation versus interpretation by using an example of something that someone from conservancy management or MEFT did which a participant did not like, or that affected their experiences with wildlife negatively.
- Homework given: Choose an incident by someone who impacts your life in relation to living with wildlife and write
  1. Your observation, your interpretation, your feelings, your universal needs in relation to incident
  2. The feelings and universal needs of the other person
  3. Propose strategies where all universal needs could be met

NVC concepts and ideas for the lesson:

Observations versus interpretations:

1. Observations are the information we can receive from our sense of sight, touch, hearing, taste, and smell.
2. Interpretations, including judgments, are the meaning we give to those observations to make sense of the world

Outcomes Conservancy 1

End of lesson

- example of negative interaction with manager led to understanding of what leadership skills are necessary, including showing respect in interactions with community
- example of problems with the PH - that he did not pay up or do development projects for community

After one week:

- reported examples of applying nonviolent communication in various contexts e.g avoided conflict in a labour dispute, in an incident with a drunk person, and mediated in a family conflict
- reported examples of showing consideration and empathy to wildlife, e.g seeing a zebra calf being treated with care, not hunting a duiker and tolerating squirrels in the homestead courtyard.
- shared NVC principles of empathy and tolerance with others
- reported examples of self-improvement at being more tolerant and less ill tempered
- Appreciation for learning in group and sharing stories

Outcomes Conservancy 2

Outcomes:
End of lesson

- appreciated importance of observations vs interpretations and how it can help to reduce conflict
- understood importance of listening and not interrupting and when someone says something bad one should not get angry but try to understand where the person is coming from

After one week

- reported examples of NVC use for better connection, compassion, and conflict avoidance: A participant managed to avoid conflict with friend over him owing him money, a participant had a compassionate conversation with a friend who dropped out of school
- applied concept of observation versus interpretation in a wildlife encounter - One participant had an encounter with an elephant - he heard noise in bush and decided to investigate rather than run away from fear it was a witchdoctor. He also applied the concept of wind direction to avoid the elephant encounter

Outcomes Conservancy 3
End of lesson

- **No checkout**

After one week

- appreciated specific universal needs such as respect and cooperation, noticing them in interactions around them
- appreciated the concept of empathy –that it brings tolerance and if you listen first, you are more likely to get your needs met
- noticed difference between observation and interpretation and when you see people making interpretations that are not based on observations it can cause pain- reported on how this was used to resolve a conflict over an unpaid debt.
- discussed with elders about the good and bad of living with wildlife and concluded it was better to have wildlife but needed better leadership for the conservancy

Week four: Nonviolent communication training – empathy and making requests through a role-play conversation with an elephant

Aim:<br>5. To learn about local cultural stories about different wildlife species in order to understand how or if they impact human tolerance towards different species<br>6. To learn two key NVC concept – Requests, empathy and empathic listening<br>7. To learn about a key differentiation in NVC – requests versus demands<br>8. To practice empathic listening, using an elephant in a question-and-answer role play, in order for participants to understand that animals also have feelings and universal needs
**Lesson content:**<br><br>• Homework revisions on observation versus interpretation and discussion. Examples used were; field officer comes late to report damages and compensation is not enough, meat confiscated by MEFT from poachers - MEFT said they will burn the carcass, but participants interpretation was that MEFT feasted on it themselves<br>• NVC training on request versus demand<br>• NVC training on empathy and empathic listening<br>• Role play with one facilitator as elephant and participants having a dialog with elephants asking it questions
**NVC concepts and ideas for the lesson:**<br><br>***Requests versus demands***<br><br>1. Requests are suggested strategies that are connected to needs. They are:<br>2. Specific, affirmative and doable<br>3. Unlike demands, they are made with an intention to create genuine agreement, not to be accepted by manipulation or coercion.<br>4. A "no" to a request is an opportunity to better understand and consider the needs not satisfied by the request<br><br>**Empathy**<br><br>1. Empathy is a state of being present to the experience of another being (or oneself) without judgement or trying to change that person or their experience.<br>2. Give empathy by listening silently or/and reflecting back feelings and universal needs.<br>3. Giving and receiving empathy creates connection and understanding, which moves a human receiving empathy to greater calmness, openness, creativity, and willingness to listen.<br>4. Give empathy before trying to express your opinions or offer solutions<br>5. "Giraffe" language is used to indicate communication (speaks and listens) with compassion and empathy while "jackal" language applies to judgmental or criticizing language.
**Outcomes Conservancy 1**<br>**End of lesson**

- Learnt that Universal needs apply to animals as well as humans

After one week

- Role-play with elephant was impactful - brought realization that need to keep corridors open (elders told us that but we did not listen) and apply mitigation measure because elephants are just trying to feed themselves
- awareness about empathic concern in general, a participant shared that before she did not think to bother about her friends' problems
- reported behaviour change showing empathy towards wildlife- springbok and hyena story
- realized that coexistence with wildlife was possible
- expressed hope that relations with conservancy could improve

Outcomes Conservancy 2

End of lesson

- understood difference between requests and demands and when hearing a "no" one could try to find other requests and not give up
- understood difference between empathy and sympathy

After one week

- understood that when one responds empathically to someone the other person responds nicely
- elephant role play - realized that animals are like people in that they have same feelings and needs, that elephants would also like freedom to roam freely without being chased by people

from elephant role play realized that humans are not empathic to elephants needs and maybe building in corridors is not a good thing

Outcomes Conservancy 3

Previous week was cut short due to a fire at camp, we did homework of conflict situation identifying observation, feelings, needs, of both sides of a conflict and then identified strategies to meet all needs.

Used example of MEFT reintroducing kudu into conservancy which then caused damage to crops.

End of lesson

- learnt new things from cultural stories
- appreciated difference between request and demand and how to best approach someone
- learnt to see things from elephant perspective

After one week

- reported behaviour change showing more empathy- a participant convinced some people to give a poor man food instead of being rude to him and thinking he was lazy; another helped his friend empty water from his canoe. Before workshops both would

not have bothered to intervene

**Week five: Understanding conservancy policy documents and unpacking conservancy governance issues from dialogues with guests**<br>**Aims:**<br>5. To learn about the policies that govern conservancies<br>6. To get skills and practice presenting to an audience<br>7. To ask questions and have discussions with an invited guest to learn and understand what is working and what are the challenges in the conservancy<br>8. To practice nonviolent communication skills in dialogues with the guest
Lesson<br>• Policy document presentations policy; National HWC policy, Conservancy constitution. Participants were divided into groups last week and met to prepare their presentations. Each group presented and then gets feedback on their presentation skills, followed by questions and discussion from the whole group.<br>• At 10:30 the guest arrives, and we have an ice breaker game. Then group give a short recap of what they have learnt so far in the workshops. Next the guest shares what his job is and what a typical day looks like for him/her.<br>• Then floor opens to questions from participants to the guest and answers from guest
**NVC concepts and ideas for the lesson**<br>Participants try to apply what they learnt to the questions and discussions
**Conservancy 1:**<br><br>Issues arising:<br><br>Policy documents: National HWCpolicy and conservancy constitution<br>• Participants thought that actions on ground were not as stated in the constitution<br>• compensation payments were not at the rates according to the new policy<br>• only 2 participants had heard of the new policy and none had seen the new policy<br>• questions asked:<br>why does conservancy pay less than damage, why are small species not compensated as they cause most damage, differences between old and new HWC policies, process of damage reporting-seem to be inconsistencies in application of processes, why some people get paid different amounts for same area of damage, querying incidence of potential corruption in reporting damage-people do not get paid out according to what

they claimed, why game guards come late to report damage when evidence has vanished e.g. livestock predation.

- some in group did not realize conservancy paid compensation according to national HWC policy
- guest explained that if conservancy had more money conservancy could pay for smaller /other species
- some did not know that MEFT and conservancy contribute equal amounts for compensation payments (NAD 60,000 each)
- guest agknowledged sometimes game guards do not go to investigate damage and encouraged participants to report them as they would get reprimanded

Outcomes

End of lesson

- Understood why conservancy was not going well - because the constitution is not being followed
- Appreciated that the dialogue went well with the guest and there was mutual respect between him and the group
- Appreciated understanding how compensation works
- Understood that some game guards are not skilled enough to do their jobs and that may be reason conservancy was not working well

After one week

- knowledge about which species are compensated-thought all animals were compensated for, as well as new amounts according to new policy
- thought amounts in policy were not in proportion to the importance of crops for peoples' livelihoods
- Understood that conservancy management do not follow constitution in relation to payments –they get monthly salaries while constitution says they should get sitting fees
- felt hopeful that things could be improved in conservancy with new understanding
- appreciated new knowledge from policy documents and guest, especially of compensation
- understood reasons there was insufficient money to pay compensation for small species because there are no campsites or lodges-hopeful that can change
- learnt about some new mitigation measures for elephants-e.g clearing of buffer zone around fields and using chilli bomb
- heard that game guards do awareness about the use of mitigation measures, but participants had not seen that or experienced that
- after reading policies felt that people making them do not consult locals as they are not relevant to local conditions
- enjoyed presentation by one presenter who mentioned he was in Katima and went to MEFT to get copy of HWC NP and no one there knew about it, then they gave him a copy of the old policy, then he said no there is a new policy, then they found one but said no we can't give it to you ask your conservancy, but there is none at the conservancy.

Conservancy 2

Issues arising:

- HWC policy- only 2 participants heard about the policy and 0 new there was a new policy. But student facilitator said she was at AGM when they mentioned the new policy
- Questions for guest: how are game counts done and do they count exactly or is it an estimate, controlled early burn and what about the small animals that get burned, many questions about the compensation process and criteria for making a claim
- Guest explained that some animals lose out in controlled burns, but bigger animals gain from better grazing
- problem with delay in game guards coming to report damage – a participant waited three days in 2016 and she has still not been compensated. Guest felt bad about that as it is the game guards' job to report the damage and suggested in future to get a neighbor to be witness. However, another participant felt it could be problematic if a neighbor has a grudge against you and lie. She asked if photo evidence would be considered. Guest said it would depend whether the game guards felt the evidence was credible and it was also a challenge for the guards to decide on the best available evidence whether to trust a claim or not. Facilitator recommends conservancy draw up a list of what evidence farmers should collect to ensure their claim is trusted and guest says he will take this suggestion to the office

Outcomes

- appreciated guests honesty and that he seemed to know his job well
- appreciated learning new things about damage reporting and procedure to follow
- appreciated new information about early burning, that small animals lose but big animals gain from new grazing
- thought solution offered by facilitator regarding criteria for damage evidence to make a claim of conservancy was good
- realized the importance of universal needs of cooperation and love. Game guards need to cooperate among themselves to help farmers, neighbors need to love each other to provide reliable evidence when making damage claims

After one week

- realized that maybe they do not prepare themselves enough to make damage claims and that's why game guards reject their claims
- showed empathy to game guards that maybe they are busy sometimes
- other community members expressed interest in workshops and a participant shared what they learnt about HWC and damage reporting
- realized that conflict results when universal needs are not met for both people and animals
- appreciated universal need of love and that love is needed for people to have empathy and do their job with passion
- thought everyone should get NVC training if we want people to help each other.
- A participant wanted to know how sick animals could be helped

Conservancy 3:

Issues arising

- questions asked included, how do they know how many animals are in the conservancy, what is importance of small animals like tortoises, why don't office share information with community, for e.g the constitution, does the conservancy make enough money as it seems the office does not have a working computer, what is process of reporting damage and why is compensation payment delayed, why does it sometimes take a long time for game guards to investigate damage
- was confusion around when game guards come late to report damage, guest said farmer can get someone else to witness and then when game guards comes the farmer brings the witness to testify-this information is not written anywhere
- problem of delay in compensation payments-turned out MEFT had changed rules and required extra signature from induna, but induna signed before the forms where complete, so they were waiting for MEFT officer to come again
- seems MEFT has required more stringent rules for this conservancy to claim-they require the extra signature from the induna who has to verify the claims, it seems this extra step is not required by other conservancies from MEFT, so group concluded that MEFT does not trust their conservancy
- guest said if community was not getting information from their area representatives, they should replace them
- regarding delays in reporting damage, guest said sometimes they are very busy, and they go on first come first serve-but they will prioritize predation events as they know farmer needs to skin carcass as soon as possible
- problem of MEFT changing rules and not informing conservancy in time to process claim forms
- regarding wildlife monitoring, guest explained when they do monitor, they focus on large species as those are the ones important for quota setting and that they benefit most
- group were concerned about smaller species like tortoise, guest explained they are important as tourists like them especially children

Outcomes:
End of lesson

- no checkout – facilitator asks for needs met and unmet from session

After one week

- knowledge from the constitution was useful-in once case it helped resolve conflict over meat distribution as people who were 18 years old but had not registered as members wanted to get meat but could not- the participant could explain the rules based on his new knowledge of the constitution
- knowledge from the HWC policy and guest made a participant realize that she should measure her fields as the claim is based on the size of fields
- other knowledge was useful in reducing blame of late payment by office of

compensation as guest explained the delay was from MEFT because they had changed the rules without informing the conservancy

- appreciated the guest explanations about the problems and complexities with dealing with compensation claims

Week six: Understanding conservancy policy documents and unpacking conservancy governance issues from dialogues with guests
Aims:

5. To learn about the policies that govern conservancies
6. To get skills and practice presenting to an audience
7. To ask questions and have discussions with an invited guest to learn and understand what is working and what are the challenges in the conservancy
8. To practice nonviolent communication skills in dialogues with the guest

Lesson

- Presentations of policy documents; conservancy HWC policy. Participants were divided into groups last week and met to prepare their presentations. Each group presents and then gets feedback on their presentation skills, followed by questions and discussion from the whole group.
- At 10:30 the guest arrives, and we have a ice breaker game. Then group give a short recap of what they have learnt so far in the workshops. Next the guest shares what his job is and what a typical day looks like for him/her.
- Then floor opens to questions from participants to the guest and answers from guest

NVC concepts and ideas for the lesson
Participants try to apply what they learnt to the questions and discussions

Conservancy 1:
Issues emerging

- only 4/16 in group heard about bm HWC policy,
- 0/16 have seen the policy, 16/16 think it is useful
- none in group use chili bombs
- they asked the office to photocopy copies for their group work, but the office had no paper
- questions for guest: many questions about animal behaviour and how one knows if it is about to attack, mitigation measures and who is responsible for ensuring they are

applied correctly, how do they insure game guards come on time to report damage and help with problem animals

- guest explained how to create buffer zone around fields to prevent elephant damage, how to use chili bombs, mitigation measures for small animals such as porcupine
- problem arose of inconsistent supply of chili by an NGO to the conservancy
- problem of office supplying wire and chili to farmers, but they used them for other things and not to prevent damage
- guest explained that the field officer does outreach and then goes to check if it is done correctly
- facilitator asked how people get information on mitigation measures and guest explained that people must go to office to ask, but it is the job of area representatives to ask for awareness campaigns

Outcomes:
End of lesson
no checkout, in general enjoyed the guest

After one week

- reported empathy towards wildlife – game with animal sounds made a participant notice a mouse in the grass near his house and learn about their behaviour and watch them instead of killing a mother and its young
- appreciated learning about how to behave when encountering elephant in bush-to use sand to check wind direction
- appreciated learning about different mitigation measures to prevent damage
- understood why there was a shortfall of funds in the conservancy due to PH not paying.

Conservancy 2:

Issues emerging from HWC policy document:

- only one participant knew about the existence of the HWC policy for the conservancy but had not seen it.
- A participant concluded that animals can't live in peace anymore because of people. So he asked where did people come from? The group discussed various ideas ranging from population increase, cultivating near park and river, according to bible Adam and Eve sinned and punishment was to grow their own food, because community agreed to establish the conservancy so animal numbers have increased, before it was more subsistence farming but now fields are bigger, climate change.
- most participants applied some mitigation measures - wire fence with tins, chili for elephant, whip for birds, throw fire logs at elephants, scarecrows, trenches covered in grass for porcupines who fall in and then eat meat, chili inside maize cobs for porcupine-when chili gets in eyes it gets disoriented and can't escape, then you find it and kill it.
- A participant comments that mitigation measures are cruel and does not understand if we are supposed to be conserving wildlife and what about empathy they have learned.
- mitigation measures are not always effective, e.g if you are asleep at night when

elephants come you aren't able to put chili
- suggested to have more awareness about mitigation measures involving MEFT

Issues arising from guest:

- Asked many questions: what are challenges guest faces in his job, where exactly does the money come from for the conservancy and if there is so much money why are there so many unemployed youth, what are other strategies to get income since professional hunter did not pay the conservancy, what are the rules and agreements with the hunter because how come the hunter was able to hunt on credit and then end up not paying, how are game guards appointed, through votes or interviews, in the opinion of the guest, how is the conservancy doing-is he happy with how it is going, there seem to be many different departments that do different things so is there a department that looks after sick or injured wildlife, what is relationship between conservancy staff and MEFT staff from the national park, why has the electricity project stopped
- guest expressed gratitude for the workshops and appreciated that it was for community and not staff as community don't usually volunteer to do things
- guest said there was a problem with the attitudes of the community - that when they report damage that the animals belong to the office rather than the community. Community expect office to not follow rules, for e.g when a hippo causes damage that want it killed so they can get the meat. They also don't know the rules in general
- guest explained that delay in setting up tourist lodges is from MEFT and not the office
- guest explained the income for conservancy is not enough to address unemployment
- guest explained that contract with hunter is through MEFT and not the conservancy, so it is out of the hands of the conservancy if things go wrong with the hunter's contract
- guest explained that electricity project is in phases because it is expensive, so each phase has a budget. The conservancy is waiting for more funds to continue with the next phase

Outcomes:
End of lesson

- guest expressed gratitude for workshops
- knowledge about the constitution brough clarity on how the conservancy works
- appreciated the guest for his knowledge about policies and how well he explained things
- knowledge about electricity contractor helped to not blame him- she thought he just ran out of money
- expressed some disappointment around answers about progress on development project and delays caused by MEFT for lodge approval
- a conflict in the group emerged during checkout about interruptions during dialogue with guest and we resolved it using NVC
- applied concept of observation versus interpretation to ask question

After one week

- expressed inspiration from guests' motivation to get involved in conservation and his knowledge in answering questions
- some felt hopeful that lodge problem will be resolved soon, electricity project will get finished and the conservancy will get more income
- some felt discouraged at inefficient use of funds and unfinished projects, hunter not paying
- learnt that MEFT was responsible for quota setting for wildlife hunting
- all information was very useful

Conservancy 3:

Issues emerging

- all group (11/11) did not know that conservancy HWC policy existed
- some did not know that using chili bombs was effective for elephants. Only one participant had used chili and she learnt about it from a MEFT officer she met by chance at the conservancy office. Others did not get information on using it
- 5/10 use tin fences but they tend to be ineffective after 3 or 4 times
- guests complained about community members not attending meetings, only being motivated to come if there are benefits such as food, when the come to the AGM they don't have much to contribute because they have not attended area meetings, so they don't know what's going on in the conservancy
- it emerged there was a general problem around how to get information to communities about the meetings, that when flyers are pinned onto trees, they are taken down, indunas don't pass on information, area representatives only sms a few people and expect information to reach everyone
- participants complained that area representatives don't do enough to inform community about meetings
- discussion around who gets benefits - seems there are no rules written down and conservancy makes rules as they go along. For e.g. a person who was not born in the conservancy can only become a member if they have lived there 5 years, but it seems if you do not qualify you can use a members details and ID to make claims, even if you are from another country.
- discussion around compensation for death - confusion was around under what conditions you can claim, whether you are walking at night or in a corridor you may not get compensated. Conservancy only pays for funeral costs while MEFT pays the family for the loss
- membership information is not updated as there are people on lists who have died
- guests complained people don't want to register as members but when benefits are distributed, they complain why they have not been registered
- guests felt discouraged and want to resign because of lack of attendance at meetings and being accused of various things. They felt there is a general lack of cooperation between members and area representatives
- area representatives are applying their own rules - they try to reward only those that attend meetings and trick them into coming to meeting by promising food

- there is a general problem of how to get information to 700-800 members in each area.
- guests acknowledged the only way members can get information on rules for compensation is through the area representatives

Outcomes:
End of lesson

- group were generally disappointed with the guests
- found the conservancy HWC useful and to learn that since 2015 compensation had not been paid in their conservancy
- found guests advice on mitigation measures useful, especially the issue of need to measure the field size to know how to claim
- learnt how area representatives communicate with members about meetings

After one week

- after discussion and complaints about lack of communication by area representative, one SMSed some participants about a meeting, and it was well attended. The participant felt there was now more trust between community and area representative
- records of applying empathy-a participant reported showing more empathy to her child when she wanted to eat chili, another showed empathy to a sick friend who felt lonely
- expressed surprise learning that compensation for injury or death will only be given if incident occurred in or near the village as most of the danger is by the river
- found information on mitigation measures useful

Week seven: Empathic listening practice and unpacking conservancy governance issues from dialogues with guests
Aims:

4. To practice empathic listening by guessing feelings and needs
5. To ask questions and have discussions with an invited guest to learn and understand what is working and what are the challenges in the conservancy
6. To practice nonviolent communication skills in dialogues with the guest

Lesson

- Presentation of Empathy "blocks"

- Demonstration of empathic reflection guessing feelings and needs
- Pair work practice empathic listening
- At 10:30 the guest arrives, we have an ice breaker game. Then group give a short recap of what they have learnt so far in the workshops. Next the guest shares what his job is and what a typical day looks like for him/her.
- Then floor opens to questions from participants to the guest and answers from guest

Homework-project assignment: What are you most excited to work on based on what you have learnt in the program? For. Eg. changing a policy, raising awareness, testing mitigation measures, resolving a conflict. Write as many as you want. For each idea:
Who are the stakeholders you will need to work with?
Which of the ideas you learnt about will be important?
What knowledge or skills will you need?
What resources will you need?

NVC concepts and ideas for the lesson
Empathy blocks

1. Empathy blocks are things we say that can block empathic connection. Examples include:
- Advising: “I think you should….”
- Educating: “This could turn into a positive experience if you just….”
- One-upping: “That’s nothing. Wait till you hear what happened to me.”
- Consoling: “It wasn’t your fault: you did the best you could.”
- Sympathizing: “Oh you poor thing”
- Story telling: That reminds me of something I heard on the news….”
- Shutting down: “Cheer up. Don’t feel so bad.” “Come on, lets go (for an ice cream/to the gym etc)”
- Interrogating: “ When did this begin?”
- Correcting: “That’s not how it happened.”
- Reassuring: “It will all be ok”
- Denial of feelings: “Don’t worry, Its silly to worry”
- Minimizing: “That isn’t so important”
- Diagnosing: “ Your problem is you’re a compulsive worrier”
- Analyzing: “I think you are reacting like this because of your personality type”

from cnvc.org

Conservancy 1:
Emerging issues

- no lodges or campsites have been built in the conservancy because people are living in the core wildlife area
- people are farming in the core area because of water and better soil especially during drought
- compensation is less than costs and small animals are not compensated for
- CMC get paid monthly and not per meeting as the constitution says

- no process for real community input into budget
- manager not skilled to deal with community
- suggestion that PH should pay for photos in addition to hunting
- there are no quotas for community to hunt smaller species as poaching is too high

Outcomes
End of lesson

- some felt the guest was avoidant in answering questions
- facilitator felt discussion did not go well as participants were not using communication skills in the dialogues

After one week

- Felt that management have been in position for a very long time and every year make same promises of change but nothing changes. Problems remain the same.
- But feel some hope that things can change once they learn and understand better about the conservancy through the workshops and if new leaders come in things could change

Conservancy 2:
Issues emerging

- asked questions about wildlife diseases, which ones spread to humans and livestock, how do they get transmitted, how to prevent getting them, does one treat wildlife same as livestock. Particularly interested in foot and mouth
- guest explained that foot and mouth is carried by hooved animals, but they don't get sick, only spread to cattle. Cattle don't die but they lose condition and that's why it has big economic impact. People can also eat the meat and won't get sick. Quarantine is expensive and people can't export cattle outside from the Zambezi region – that is why price is lower for cattle. Guest suggested communities set up their own abattoirs and process meat in Zambezi as processed meat from Zambezi can be sold, e.g biltong (dried meat) and sausages. He also suggested community get hunting license and set up their own trophy hunting operation instead of foreign hunters making all the money from hunting. Guest also advised participants to cook the wildlife meat very well as he is not sure all meat hunted is inspected before being distributed to community.
- asked second guest from department of agriculture about what is the recommended no. of cattle and hectares of crops to grow.

Outcomes
End of lesson:

- group expressed appreciation for the information

After one week

- participants reflected on the knowledge they had learnt about wildlife diseases showing good knowledge retention after one week
- appreciated knowledge of the guest about wildlife diseases and the issue of expense of

quarantine for the sale of meat outside the province

- appreciated suggestion by guest that community should get trained to be a professional hunter
- there was meat distribution during the week and participant expressed uncertainty about the health of the meat

Conservancy 3

Issues emerging

- the discussion was bit difficult because the guest had only been in the job for 4 months and only attended 4 meetings
- it was difficult to get information from the guest as the answers were short
- struggled to determine exactly what the job of chair and vice chair were, but it seems it is oversight
- the guest is only called to some meetings and is not concerned which meetings she attends as she trusts she is called when necessary. She does not want to attend too many meetings as she has other things to do. Participants were concerned that she would be missing important information which will not allow her to do her job well. The group requested that she ask which meetings she is not invited to and why, but she was not prepared to do that
- participants asked if she received training for the job and she said no
- it seems there is a general lack of clarity on what the role of chair and vice chair are and what exactly is the process of how they hold the office accountable- constitutions is also not clear on that
- in general participants were disappointed with the guest and felt she was not dedicated or passionate about her job and was not interested in learning about her role

Outcomes
End of lesson

- the group gave the guest empathy when she expressed, she was feeling sad that they laughed at her for not being able to respond to questions since she had only been at job for 4 months. The empathy was not exactly NVC but she was happy with the empathy given

After one week

- reported examples of responding empathically to people in various situations: e.g a participant responded with empathy and understanding with a sibling who borrowed her laptop without asking, a participant responded with empathy and understanding to a friend who owed him money, a participant responded with empathy and understanding towards children at church who were disrupting the service.
- appreciated learning that the conservancy were creating cutlines to count wildlife

Week eight: To practice using nonviolent communication concepts and unpack conservancy governance issues during dialogues with guests
Aims:

4. To practice using nonviolent communication concepts
5. To ask questions and have discussions with an invited guest to learn and understand what is working and what are the challenges in the conservancy
6. To practice nonviolent communication skills in dialogues with the guest

Lesson content

- Explain concepts of "jackal" and "giraffe" language – terms used to reflect judgement (jackal) and empathic (giraffe) language
- NVC practice responding to a difficult message using observations/feelings/needs/requests to convert a "jackal language" conversation to "giraffe" language using example of Elsa and the manager:

Elsa:" Where has all the money gone in the conservancy? Why are you people all so corrupt?"
Manager: "You members are so lazy and useless. You are not prepared to come to meetings and be informed of what we do and then you complain that we don't do anything and are corrupt!"

Conservancy 1

Issues emerging

- policy documents are not made available to community members, so they don't know how the conservancy is supposed to operate. Also, it doesn't get updated to reflect any changes agreed on so not everybody knows latest rules.
- the conservancy lost NAD 1.7 million last year due to the PH not paying and there was no feasible way to hold him accountable. Even if funds were available to prosecute, money would have gone to MEFT as they are legal holders of the contract with the PH
- indunas control information flow in conservancy - they only allow area representatives to speak when induna calls a meeting because they worry the area representatives will take over control, so not all information is getting through to community
- CMC don't have will power to follow constitution in face of community pressure – e.g when salary increase for game guards was voted on at AGM
- conservancies ask for many social development projects from the PH - if it's too much and the PH can't deliver there is no way to hold him accountable and he can use it as an excuse to extend his contract-that's why PH got a 6 year contract instead of 3 years. This was negotiated by MEFT and not conservancy

- no fire management plan or strategy or outreach – when veld gets burnt PH complains there are too few animals in area to hunt

Outcomes:
End of lesson

- appreciated skills, knowledge, openness, and honesty of the guest
- understood what needs to be done to improve conservancy and felt inspired to move forward on that
- new knowledge increased trust for conservancy management
- realized problem of lack of communication by the office to community and control of knowledge by indunas so not everyone is heard – suggested group be called to meetings at office
- guest expressed gratitude to group and their interest showed love for the conservancy
- guest expressed gratitude to the research team and format as tool for understanding and dialogue - was apprehensive at first about the research
- guest made suggestions on how the group could be involved in the future of the conservancy

After one week

- No check in from last week – NVC exam

Conservancy 2

Issues emerging:

- Questions asked: why were community not informed about the new professional hunter, what are the development projects in the conservancy, why has a permanent chairman not been elected since 2018, why was a chairman appointed who already has a job in Katima (why was someone unemployed not given a chance), how does hunting protect animals and isn't there another way to get income for the conservancy, what are the challenges of the CMC and what are solutions, when will electricity reach the villages and will it reach all the houses,
- There were many delays in finding a suitable investor to develop the campsite and choice of camp site, when a site was selected it was further delayed because of a land dispute, then investors lost interest. When it went out to tender again only 2 people applied but they were not suitable because they did not offer sufficient social development projects. Have been trying since 2014.
  - money was invested in two "white elephant" projects - a traditional village for tourists and a craft project were never completed and then it was decided to do the electricity infrastructure project instead
  - a corruption trial of the previous chairman has been ongoing since 2018. Without an outcome a new chair cannot be elected
  - issue of sustainability of hunting and international pressure against trophy hunting was discussed

- challenges for CMC included lack of skills of area representatives such as that they can't write, they don't want to speak in public, they don't report back to community, they can't write reports, so office has no information for their reports, they also give confidential information to community before it is ready to go out. A request was made to the group to please select area representatives with skills to do their job
- it was explained that households will need to pay for electricity connection to their house as conservancy is only paying to bring the transformers to each village. Participants were not aware of this

Outcomes:
End of lesson

- appreciated learning about the issues around delays in getting the lodges and that chairman is elected at AGM

After one week

- no checkin – NVC exam

Conservancy 3

Issues arising:

- asked similar questions about compensation and citizenship as they asked the guest last week, such as number of years lived in conservancy before getting benefits and location when injury or death. This guest who was higher ranking than the guests from last week gave different answers, highlighting the lack of availability of accurate information and policy confusion. This resulted in participants thinking the previous guest's inadequacy in conveying correct information and the present guest complaining that the previous guests were not qualified to do their job. Some were not able or willing to speak in public. They were being chosen for the benefits they could bring to their immediate family and not based on skills
- guest suggests that the facilitator organize a training program for area representatives
- policy says people will only get compensated if they use mitigation measures or do not provoke an animal or go to areas where they know there are wildlife-e.g they should only go to the river where there is a crocodile proof enclosure
- information about who contributes to compensation funds was discussed-MEFT and conservancy contribute equal amounts. But for death and injury conservancy pays towards funeral but MEFT pays for injury and death according to national HWC policy
- facilitator asked question about compensation for small species - whether conservancy discussed this option. Guest reported that the community decided it was more of a priority to fund bursaries and funerals
- issue of delay in paying damage compensation- since 2015 conservancy did not have enough money in previous years as hunter was not able to hunt according to quota for elephants-quota was for 3 but hunter only found 1. Now they have funds so they will backpay all the claims
- question was asked about what the use of permits was is if people collected natural resources without permits e.g reeds without a permit. Guest explained that game guards

cannot see everything –that community buys meat from poached wildlife so they also have responsibility

- asked if people get injured or killed while collecting without a permit would they still get compensation – guest replied yes, they will because MEFT feel they have to show empathy for the family's loss

Outcomes:
End of lesson

- no checkout

After one week

- no checkin – NVC exam

Week nine: World café on group projects, nonviolent communication assessment, final reflections and program certificates
Aims:

4. To evaluate participants knowledge of nonviolent communication
5. To discuss project ideas for the groups to implement post learning program
6. To celebrate end of program with presentation of certificates of participation

Lesson

- NVC written evaluation

World café to discuss projects: a station is created for each project idea. Participants move around and spend 10 minutes at each station discussing ideas for the project. After everyone has visited each station, everyone selects a station/project they would like to implement. Each group then discusses further and reports back to whole group. The project stations were based on the issues identified by the first author R.K: Projects suggested by Ruth

- Mitigation measures
- Compensation
- Constitution and other policies
- Communication
- Benefit distribution
- Natural resource management

- Final checkout
- Certificate ceremony

Conservancy 1
Project ideas proposed by participants

- mitigation measures and animal behaviour awareness project
- fire management awareness project

Issues arising from world café discussion on projects

- benefits are few, members used to get cash payments but that stopped due to MEFT directive, NAD 60,000 goes to traditional authority
- meat is not distributed equally between areas
- compensation for wildlife damage should cover all costs
- communities are not consulted adequately when policies change, policies do not reflect realities on ground and take a long time to change

Final checkout

- general appreciation for program – helped to improve coexistence with wildlife
- grateful to conservancy management for allowing the workshop – it improved relationship and understanding with management
- appreciated communication skills – not just in context of wildlife but also improving relations with family and neighbors' e.g., when cattle go into neighbors' fields
- appreciated NVC training; how universal needs helps connect people, help resolve conflicts, that animals also have universal needs and that understanding increases respect for animals
- better understanding of importance of zonation plans and wildlife areas for campsites and lodges to increase money for compensation of damage
- expressed hope that group will pass on knowledge to community to resolve conflicts and be active in solving problems in conservancy

Conservancy 2
Project ideas proposed by participants

- butchery project to produce processed meat as suggested by the vet, to solve the problem of meat export from Zambezi
- meat health project
- HWC mitigation project
- participants form an advisory group for conservancy management to share information they learnt from the workshops. They also start an information center for members
- rubbish management and recycling
- a project to ensure selection of competent area representatives

Final checkout:

- general appreciation from workshops – received hook and not just fish, feel privileged to have received knowledge and skills (three participants)
- used NVC skills and concepts – used observation to understand why cattle are coming into his yard, then used improved communication skills to resolve conflict with neighbor over cattle (one particpant), making requests rather than demands and able to find solutions to meet everyone's needs (one participant),
- empathy towards wildlife – empathy enables one to think of conserving rather than destroying wildlife, thinking about small animals that get destroyed during fires, general love towards animals, understanding of impacts of humans on wildlife
- empathy towards people – now feels general love towards people, appreciate learning from group and better sharing of food in group 9at the start of the workshops everyone took for themselves without ensuring others also got food)

Conservancy 3

Project ideas proposed by participants

- farming guinea fowl
- gardening project

Issues arising from world café discussion on projects

- there is a lack of clarity and agreement on staff policy and length of employment/elected service. Community feels people stay in their jobs for too long
- compensation is not enough to cover costs
- communication between area representatives and community is poor
- people do not have knowledge of mitigation measures and how to behave when encountering wildlife

Final checkout:

- general appreciation for workshops
- reported examples of behavior change towards increased empathy and understanding towards wildlife – a participant stopped hunting opportunistically with his dogs, others understood that coexistence was possible
- reported examples of behavior change towards increased empathy and listening towards people – appreciation for skills which have improved communication and reduced conflicts (two participants), ability to mediate conflicts (one participant), treating children with more compassion and not beating them (one participant), respecting different viewpoints (one participant), better relationship with brothers (one participant), better tolerance to others (one participant), trying to meet others needs whenever possible (one participant)
- changed cultural values - seeing wife as more equal (one participant)
- better understanding of conservancy – more appreciation but better leadership and skills are needed (two participants)
- appreciated universal needs vs strategies – that money and cars are strategies to meet universal needs (one participant)

- six participants reported changes in their personality towards being more agreeable and open to different opinions and learning from each other
- better understanding of MEFT

Table S3. Summary of notes from group interviews after three years. CMC=conservancy management committee, HWC=human wildlife conflict, NVC=nonviolent communication, MEFT=ministry of forestry and tourism, universal needs=universal human needs, PH=professional hunter

Evaluation three years later – July 2022

Conservancy 1

- reported examples of implementing mitigation measures and have less damage – a participant grew chili in her garden and bought wire for fencing from the sale of her maize. She also helps her neighbors with chili from her garden. Another participant uses vuvuzelas with elephants, and it helps, another helped his brother on his farm to grow chili and he uses it and it works
- organized as a group and did outreach on animal behaviour and mitigation measures in conjunction with game guards
- outreach group used NVC to convince people to buy their own wire for fencing as conservancy only gave small amounts of wire based on peoples' connections and status and not on need
- a participant convinced 10 neighbors to use mitigation measures and they all do something based on their ability to get resources and they are happier because at least they harvest some food now
- a participant advised her neighbors to put chip packets around the watermelon fields and the noise at night scares the jackals/foxes, so they were able to harvest more watermelon. They are grateful of her for the training she gave them.
- a participant trained his community around clearing 5 meters around fields
- game guards are also thankful to the group for doing the outreach as it has lessened their work and people are less angry with them
- the group had weekly meetings at the conservancy office to plan a chili growing project, but the project died out as they did not get the support they had hoped from the office. It was not clear why the office did not support them, the group felt maybe office staff were worried they wanted their jobs. One of guests from the program was very supportive and asked the office to support them and reminded them to put the project on the agenda for the AGM but they did not.

- the group organized a fire warning system – when they see a fire in the bush they mobilize to fight it. In the past they would have let it come close to the village, before 2019 a fire even burnt down the khuta because they did not try to stop it
- a participant did outreach about the dangers of fires for snakes
- a participant continues to have empathy for wildlife – he prevented wild dogs from taking a zebra. In past he would have allowed that and taken the zebra meat. When Ruth asked what about food for wild dogs, he said he has too much empathy for the zebra. A participant requested a man who was about to shoot a guinea fowl with chicks to not do that and have empathy for the chicks who would die without the mother and the man didn't shoot the mother.
- empathy is still used towards people: a participant is still a nice person, before she was very aggressive. Her friend in the group agreed she is much nicer now
- a participant has become a mediator at the traditional authority justice department where community members bring their disputes. He advised 5 children who took away their fathers' cattle and house because he remarried, to give him back the house and have empathy for the old man, which they did

Conservancy 2

- Reported using knowledge of animal behavior to prevent dangerous animal encounters, e.g when encountering an elephant or buffalo keeping still and not running away and then looking at wind direction and moving in opposite direction, (3 participants), using chili smoke in correct wind direction to scare elephants while camping out at night to collect reeds (1 participant). One participant told a story of friends not listening to his advice when encountering a hippo which nearly ended in disaster. One participant told story of how she was very fearful of elephants before the 2019 workshop but during reed collection in 2020 she and friends came across an elephant at river which did not see them as wind was blowing away from them. She advised the friends to stay calm, but they ran anyway and came across other elephants and ran back to her. Then she advised them to go east upstream from wind which they did, and all was well. Now they listen to her advice. Another participant's brother died in 2020 from an elephant while collecting devil claw- she lamented if he had attended the workshop maybe he would have known how to behave and not get killed.
- Knowledge of how the conservancy works helped a participant to get a job in the conservancy
- Reported changes in empathic concern that have persisted since 2019 training - a participant reported that before 2019 workshop she would get very angry with people and respond aggressively or sulk, even for one month she would not speak to her husband if he made her angry. After the workshops she changed and now listens first, can forgive more easily, and understands need to communicate and find solutions to problems. Another participant reported that empathy is still in her personality now, she gives people money to get to clinic and credit in her business. It does seem that more people come to buy from her now
- Ruth asked them if they were more involved in the conservancy. They said not really because there is no point. At meetings everyone quarrels, a participant reported she did not get compensation when her fields were damaged – game guard said she should

have slept in field, but she can't always do that because her husband is often away and other people who don't sleep in the field do get compensation.

Conservancy 3

- One participant grew chili in his garden, but he didn't have anyone to sell it to and the conservancy did not show interest. Others in group asked why he did not inform them as they would have helped and bought some
- After 2019 the group did try and meet a few times but then covid came. They wanted to start a chili project but there were no funds from the conservancy because even the funds dried up for conservancy due to covid.
- Four of the group formed a group and planned to do mitigation measures outreach. They asked the conservancy office for some funds for transport and food, but office did not have and due to restrictions of gatherings due to covid they could not do it
- Personality changes and empowerment – one participant reported that before 2019 he was violent to his wife and had many girlfriends but now, he is a changed man and only has one girlfriend. Two other participants noticed his changed behavior. Another participant reported his more agreeable personality had persisted since 2019 and people have noticed this at meetings. Another participant reported still being more kind & honest and uses empathy. E.g., Before if kids did something wrong, she would beat the child but after learning about empathy she doesn't do that, she just talks to him nicely. Another participant got a job in marketing. She felt the workshops gave her more confidence and that's how she got the job. She also uses NVC to deal with conflict with her boss and that has helped her to be valued by her boss. Another participant continues to be agreeable in nature since the training as before she was short tempered. Another participant was short tempered and used to beat her kids but now she has empathy. Another participant feels he got a job due to his improved communication skills and confidence in speaking. When his supervisor got ill he acted as the supervisor for 1 year. Another participant was ill tempered before the workshop but now he is called to mediate conflicts in his village. Another participant helped his friend market insurance policies and when the friend saw how well he engaged with people he offered him a job and to get trained. He uses empathy in his shop and that has also increased the number of customers.
- Leadership positions because of training – a participant felt training helped him get elected as area representative as community saw he attended the training and had leadership skills. He developed a garden and employed local people, so they saw he cared about others and himself to develop a campsite and garden. A participant reported that his community now appoints him for many leadership roles – on the water board, school, political party. He has not been elected in conservancy as area representative as there have been the same ones for 15 years. A participant has been active in conservancy meetings and suggested to reduce tenure of office bearers to give youth a chance, but he has not been successful. He is very vocal and attends all meetings. He isn't scared to challenge management and asks difficult questions, so he is speaking for the community, and they appreciate that. He has also been elected to other leadership roles in his community. A participant has been appointed to many leadership roles because of her change in behaviour – she has been a school board

member and netball coach, at church is deacon (collecting money). A participant has been chosen as secretary at church. A participant has been active in conservancy and regularly gets called by conservancy office to advise them, also in his community

- Outreach mitigation measures – five participants reported advising their community about mitigation measures and implementing mitigation measures themselves, which has increased their yields. E.g., building a fence for hippo that helped to increase harvest, a participant reported using fencing, tins and chip packets against duiker. A participant used his knowledge of hippo behavior to instruct boat driver how to behave when encountering hippo on river.

Table S4. Attendance of workshops showing conservancy name. number and percentage of workshops each participant attended.

Conserv. name	n	Nine	%	Eight	%	Seven	%	>Six	%	Avg %
A	16	7	43.8	3	18.8	3	18.8	3	18.8	83
B	19	5	26.3	10	52.6	1	5.3	3	15.8	83.6
C	19	5	26.3	4	21.1	7	36.8	3	15.8	91.9
Total	54	17	31.5	17	31.5	11	20.4	9	16.7	83

Coding tree : definitions, categories and results

The coding tree consisted of nine broad categories: 1. *Appreciation* – records where participants expressed gratitude for the workshops or any specific component of the workshop; 2. *Knowledge and understanding* – records that showed new knowledge or better understanding of an issue or topic; 3. *Questions* – records of the questions that participants asked of invited guests; 4. *Working* – records of what is working in the conservancy 5. *Problems* – records of what is not working well in conservancies; 6. *Attitude change* – records that reflected how a person's thinking, beliefs, or intention to act towards a psychological object changed towards being more favourable (a psychological object being any discernible aspect of an individual's world, including an object, a person, an issue or a behaviour (Fishbein and Ajzen, 2010); 7. *Behaviour change* – records of actual changes in behaviour, often compared to how the person would have behaved before attending the workshops; 8. *Solutions* – records of ideas that participants suggested to solve specific problems; 9. *Social learning* – records where

participants reported sharing ideas or knowledge with people not attending the workshops. Additionally, to increase sensitivity to the local socio-cultural context we included sub-categories of the most prevalent coding themes within a category (Babbie and Mouton 2007). Thus, our results contain conceptualizations specific to the case study. Sub-categories were defined as discrete meaning units (Mayring, 2008). During data analysis we aimed at preserving the qualitative character of the workshop discussions, resulting in sub-categories being at different levels of abstraction. Coding was done by the first author and included discussions on the classification of codes into sub-categories with other experts in the field to increase reliability of the coding process.

1. *Appreciation* (n=105, 10%) - these could be categorised into seven types of appreciation (Table X), the most common being *general* appreciations for the workshop and its organizers (n=49) and for the *NVC* training, or for specific concepts or ideas of NVC (n=25). Appreciation for *social learning* was also expressed (n=12), for better understanding of *wildlife* and coexistence (n=8) and Conservancy (n=4); Mitigation measures (n=3); Knowledge and understanding leads to reduced conflict (n=3) (Table 1).

2. Knowledge and learning (n=148, 14.1%) could be divided into eight topics. The most frequent topics were knowledge and understanding about *Non Violent Communication* (n=53) and about *wildlife* behaviour (n=38). Other topics were about the *compensation* program (n=18), the *conservancy* (n=18), *mitigation measures* (n=11) and the *development* and benefits from the conservancy (n=10) (Table 2).

3. Questions (n=120, 11.4%) - eleven different question topics were coded. The most frequent topics were about the *compensation* program (n=33), for example, about processes and criteria around making claims, why it was not possible to claim for small species, why compensation amounts did not cover the full cost of losses, why game guards did not come to report damages on time or sometimes not at all. The *wildlife* behavior (n=21) category included questions around why animals behave in a certain way and how one should behave when encountering

dangerous species such as elephant, lion and buffalo. Other questions were on how species were counted and wildlife diseases and how to treat them. Questions on the topic of different *policies* (n=16) and processes included who wrote the constitution, how are CMC members appointed and what training do they get, what are criteria for membership in the conservancy and what is the process of certification for developments in core area? Some *development* code (n=12) questions were about what benefits the conservancy brings, why are developments projects delayed and taking so long such as electricity and lodges, and are there hunting quotas for locals to hunt? *Accountability* (n=9) questions included why people don't get find for collecting reeds without a permit, why appointments and salaries are not implemented according to the constitution and why the professional hunter ended up getting away without paying the conservancy? *Mitigation measures* (n=8) questions were mostly around the specifics of which ones to use, how to apply them, whose job it is to ensure they are applied correctly and how to ensure accountability in the use of materials supplied by the conservancy; *Communication* (n=7) questions included why community were not informed about various issues or did not receive policy documents, for example, that back payments for compensation would be paid, that there was a new professional hunter, and what communication strategy did they use to try get people to move out of core area. Other question codes were around the *Budget* (n=6), general *Conservancy* (n=4), *Agriculture* (n=2) and *Fire management* (n=2) (Table 3).

4. Working (n= 83, 7.9%) - records of things that were positive or working in the conservancy and these were around three topics; *Tangible benefits* (n=38), *Intangible benefits* (n=29) and *mitigation measures* (n=16) (Table 4). A detailed analysis of this category is found in Kansky 2022.

5. P*roblem* (n=109, 38.9%) –the most frequent problem type were the *Costs* (n=86) of living in the conservancy with wildlife. *Communication* (n=72) was the second most frequently recorded problem. *Accountability* (n=53) issues were the third most frequent problem. *Development* (n=49), *Mitigation measures* (n=53), *Compensation* (n=41), *Zonation* (n=18), *Skills* (n=15), *Support* (n=14), *Devolution* (n=5) and *Wildlife* (n=3) were the remaining sub categories (Table 5). A detailed analysis of this category is found in Kansky 2022.

6. A*ttitude change* (n=44, 4.2%) – there were six subcategories: *Empathy* (n=15); *Empowerment* (n=12); *Zonation* (n=7); *Coexistence* (n=5); *Communication* (n=3); *Mitigation measures* (n=1); *Fire management* (n=1) (Table 6). A detailed analysis of this category is found in Kansky & Maassarani 2022.

7. *Behaviour change* (n=79, 7.5%) – there were nine sub categories as follows: *Empathy* (n=45); *NVC* (n=14); *Empowerment* (n=10); *Animal behaviour* (n=6); *Communication skills* (n=2); *Fire management* (n=1); *Accountability* (n=1) (Table 7). A detailed analysis of this category is found in Kansky & Maassarani 2022.

8. Solution (n=38, 3.6%) - solutions proposed are reported in table S4.

9. S*ocial learning* (n=25, 2.4%) – Records where participants reported sharing information from workshops or their intention to share are reported in Table S5.

Table S5. Appreciation code - expressions of appreciation or gratitude of an object, issue, topic, person or organization

	Sub category 1	Freq.	%	Description	*Example*
1	General	49	47,12	General appreciation of workshops and people involved in workshops	*M48: I am very thankful. I'm saying this because like the skills I've acquired now. Its like not just giving a child a fish but giving a child a hook and showing this is how you fish from the river.*
2	Non violent communication	25	24,04	Appreciation of NVC training or specific NVC concepts or ideas	*F18: The concept of the human universal needs and the feelings it's a concept that feels very helpful and that helps us live better with people around us and including animals*
3	Social learning	12	11,54	Appreciation for learning from others in group	*F28: Being in a group while learning, we're learning from one another… Having love towards one another or towards things, I have love towards wildlife and all that is around us through the narration of stories from all my colleagues*

4	Wildlife	8	7,69	Appreciation for better understanding of wildlife and coexistence	*F24: We are thankful ….we learnt a lot, a lot of issues regarding the coexistence between humans and wild animals. Thank you.*
5	Conservancy	4	3,85	Appreciation for conservancy	*M34: Thanks to the conservancy office for allowing the SL program and for seeing the need for us to gain additional knowledge, even if we have had issues here and there but they have shown that love to us. I also want to give thanks to the chief of the research program, there is that light of a candle, at least when you light that little candle it will buy a bigger candle.*
6	Mitigation measures	3	2,88	Appreciation for better understanding around mitigation measures	*F5: We discussed the mitigation measures, tying things around your field and the use of chilly bombs. Those things are very useful*
7	Knowledge and understanding leads to reduced conflict	3	2,88	Acknowledgment that the new clarity had lead to reduced conflict and increased trust	*F28: This has been our complaint that the office should visit us so we understand theses issues so we don't blame them. We thank him that he is among us and also came here and shares the things we don't know. We are thankful for explaining what is going on in his office.*
	Total	105	100		

Table S6. Knowledge and understanding

Expression of understanding or knowledge or learning of an issue or topic.

	Sub category 1			Description	*Sub category 2*
1.	2. NVC	53	35,81	Issues relating to the training in Non Violent Communication	*M2:We learnt about the concept of universal human needs and feelings. The three C's (car, cellphone and cash) I thought they were all universal human needs, I thought they were vital, meanwhile they are just strategies that can help one do certain things.*
3.	4. Wildlife	38	25,68	Issues relating to animals species occurring in the conservancy	*M35: We learnt that foot and mouth disease is mostly found with Buffalo. So most of our cattle can contract the disease when they get*

					mixed up with the buffaloes. But it's not just the Buffalo but also those that have hooves are the ones that contract the disease. The vet also said that the disease is air born and that even cattle that is within a 50 km radius, or the animals around that area, will all be affected by the foot and mouth disease.
5.	6. Compensation	18	12,16	Issues relating to the compensation program from wildlife damages	*M46: So like the issue of when your cattle have been attacked by wildlife, lets say a lion has eaten one of your cows. I have learnt about the procedure of where to go, like I did not know there are payments you can get when you have that damage. So now I know the procedure and what to do about it.*
7.	8. Conservancy	18	12,16	Issues relating to the conservancy in general	*F28: The elephants, it is also their corridor, where ever they want to go they just pass through our fields. The elders used to tell us but we never used to pay attention to this. For the lesson that we learned about last week about someone acting like an elephant, we really saw that actually that is why they are troubling us. Now we are able to tell others to say why we are troubled by the elephants because it seems we are settling in their area so that is why they pass through and why they are busy troubling us.*
9.	10. Mitigation measures	11	7,43	Issues relating to mitigation measures to prevent wildlife impacts	*F33: Concerning the mitigation measures we can apply to our fields, a field officer told us that for small species you lower the wire fence and larger species you put it a bit higher, 5 meters. We should also create a buffer area for our fields and use chilly bombs for elephants.*
11	12. Development	10	6,76	Issues relating to development projects and benefits as a conservancy member	*M2: I learnt that we depend only on one thing, just elephants. Us who are not leaders we are basically blaming our conservancy that they are misusing funds but actually the resources that we have are not enough. So the same applies if I was doing that in my household and my children cry that they need me to feed them but meanwhile I don't have anything to feed them so the next day the children will go and steal and become thieves. So that's the type of concept I got.*
	Total	148	*100*		

Table S7. Questions - The questions asked by workshop participants to guests that were invited to a workshop. Guests were mostly from conservancy management committee (CMC).

	Sub category 2	Freq	%		
1.	Compensation	33	27,50	Questions relating to the compensation program from wildlife damages	*F17: Why are smaller species not on the list for compensation?*
2.	Wildlife	21	17,50	Questions about animals	
3.	Policy	16	13,33	Questions relating to the rules and processes that govern the conservancy	*M16: Who made the constitution?*
4.	Development	12	10,00	Questions relating to development projects and benefits as a conservancy member	*M21: Why have campsites and lodges not been established yet so we can use that money to compensate for small species?*
5.	Accountability	9	7,50	Questions relating to lack of trust, lack of transparency, lack of applying rules and policies or illegal behaviour	*M1: The constitution says members harvesting illegally must be fined so why are people still harvesting illegally?*
6.	Mitigation measures	8	6,67	Questions relating to mitigation measures to prevent wildlife impacts	*M34: Does the field officer go with farmers to put up mitigation measures to make sure it is done correctly? Who is supposed to make sure it is used correctly?*
7.	Communication	7	5,83	Questions relating to information flow between different stakeholders in relation to the conservancy	*F23: Why are the policy documents not given to conservancy members? It's important for them to know how the conservancy works.*
8.	Budget	6	5,00	Questions relating to how money is allocated and spend	*F43: Where exactly does the money come from for the conservancy, and if there is so much money, why are there so many unemployed youth in the conservancy?*
9.	Conservancy	4	3,33	Question relating to the conservancy in general, that did not fit into any of the other categories	*M48: What challenges do the CMC face and what ideas for solutions do they have?*
10.	Agriculture	2	1,67	Question to the agricultural officer relating to farming	*M36: What is the recommended number of cattle and hectares for crops that a farmer should have?*
11.	Fire management	2	1,67	Questions about burning of vegetation or fire management	*M36: When you do early burning what about the small animals-do you collect them first and move them before you burn?*
	Total	120	100		

Table S8. Working

Identification of issues that were perceived as positive in relation to the conservancy and living with wildlife.

	Main code	Freq	%	Description	Example
1.	Benefit Tangible	38	45.78	The monetary or concrete things people get from the conservancy	*Munk: If it is a trophy animal, it is about 180 000. From that 180 000, there is also money given for funeral cover, we also give benefits to students. We also take 60 000 for the festival ceremony.*
2.	Benefit Intangible	29	34.9	The non-monetary positive things that people get from living with wildlife or the conservancy	*UNK: When you live with wildlife near you there's that beauty*
3	Mitigation measures	16	19.27	Measures used to prevent negative impacts from wildlife that were working	*F32: I reported to the office I needed assistance, then the game guards, they came to my field about one to two weeks just trying to guard my field*
	Total	83	100		

Table S9. Problems

Identification of issues that were perceived as negative or not working in relation to the conservancy and living with wildlife.

	Sub category 3	n	%	Description	*Example*
1	Cost	86	20,92	Issues relating to the negative impacts of living with wildlife in a conservancy, both tangible and intangible.	*Unk F: crocodile are bad and when they see a human all it sees is meat. I was once almost caught by one when I was in kavango while we were swimming to cross the river. She felt bad, sad and anger, fear very that I was almost gone for good. Safety is important*
2	Communication	72	17,52	Issues relating to information flow between different stakeholders in relation to the conservancy	*M57: On Saturday we went to our community and found out they were busy distributing meat, we didn't know if that meat was inspected or not*
3	Accountability	53	12,90	Issues relating to lack of trust, lack of transparency, lack of applying rules and policies or illegal behaviour	*F32: Because the problem we have with the area rep and indunas is that they choose who goes to the meeting, it is not us who choose*

4	Development	49	11,92	Issues relating to development projects and benefits as a conservancy member	*M1: With the fish pond project there were a lot of people, so everyone was fighting to be the manager or to have a position for that project so in the end it was cancelled. So I feel its better to do private projects*
5	Mitigation measures	53	12,90	Issues relating to mitigation measures to prevent wildlife impacts	*unk: in the beginning we used to use the flash lights for the hippos and the elephants but as times went by it seems they got used to it*
6	Compensation	41	9,98	Issues relating to the compensation program for wildlife damages	*M9: if a cow is killed the conservancy people will just give you one thousand dollars and you bought the cow for five thousand, so they should try a little bit harder when it comes to compensating*
7	Zonation	18	4,38	Issues relating to spatial planning within conservancy	*unk: So you see in the near future the conservancy will no longer exist. It seems people are building mostly where there is animal corridors.*
8	Skills	15	3,65	Issues relating to lack of knowledge or skills in relation to conservancy management and governance	*Munk: most area representatives that are chosen don't know how to write. The area representatives are the drivers of the conservancy, now if they choose people who don't know anything it will be difficult for them to drive anything*
9	Support	14	3,41	Issues relating to lack of support for the conservancy or its members	*Funk: We feel the minister should also come here so that we discuss with him because the few people who we report to sit on our complaints, they don’t work and find solutions to our problems.*
10	Devolution	5	1,22	Issues relating to the national government influence on conservancy governance or management	*No short quotes*
11	Wildlife	3	0,73	Issues in relation to wildlife in the conservancy	*Munk:in 2017 there was a big meeting that happened in South Africa. That issue about why is Namibia doing hunting now while other countries have banned it, I don't know if that meeting is still going on now. To rely so much on hunting will not be beneficial,*
	Total	409	100		

Table S10. Attitude change

Feedback that reflected how a persons thinking or beliefs changed, or an intention to change behaviour

	Sub category 2	Freq	%	Description	Example
1.	Empathy	15	34,09	Descriptions showing feelings of care, compassion, perspective taking, mediating, preventing or resolving conflict	*M41: these lessons have actually changed my thoughts and ideas. Back then in the past where I use to think our thoughts was more of destroying than taking care or than conserving. So now we also know that wild animals are also beings even those animals have their own rights. For me now to see an animal I have different thoughts in the way it was earlier*
2.	Empowerment	12	27,27	Stories that show how participants felt a sense of autonomy and self-determination as a result of the workshops	*M41:For me I see that this program has changed me with a lot of issues. Like now the belief I have now if I'm talking to someone and let's say we have a conflict… the way I was earlier I was easily short tempered but now I have a different perspective".*
3.	Zonation	7	15,91	Attitude change in relation to complying with spatial plans of conservancy	*M30: if you provide water for our livestock, we'll move from there and you'll see all this, these are all fields here, so people have fields here. So we'll move from there.*
4.	Coexistence	5	11,36	Attitude change in relation to positively living with wildlife	*M29: For me ever since we started the program here where we are right now. For me now I have understood that there's actually a better way we can live together with wildlife."*
5.	Communication	3	6,82	Issues relating to information flow between different stakeholders in relation to the conservancy	*M16: So when you have damage it's not that you go there annoyed to the office but you also try to compose yourself as you go and report to the office, that also helps."*

6.	Mitigation measures	1	2,27	Issues relating to mitigation measures to prevent wildlife impacts	*F31: us farmers should take extra care of our fields and we should also fence up our fields so that we lessen the damage*
7.	Fire management	1	2,27	Issues in relation to fire management in the conservancy	*M42: The most important thing that I've learnt, the issue of just burning fire. The idea as humans to be just burning anyhow - there are these smaller species that also suffer the consequences so you should be careful of those smaller animals.*
	Total	44	100		

Table S11. Behaviour change

Feedback on an incident or story that reflected a change in behaviour compared to how a person would have behaved in the past

	Sub category 3	Freq	%	Description Narratives expressing……	Example
1.	Empathy	45	56,96	…feelings of care, compassion, perspective taking, mediating, preventing or resolving conflict towards people or animals	*M15:When I started this program I learnt a lot of things. Like whenever I was at home when I saw a common duiker just passing around, I would chase it with my dogs. But when I started attending this workshop, I was now blaming myself to say what I used to do-chasing those animals with dogs, that was a bad idea. So I have learnt from doctor Ruth how humans should live with wildlife. Like its not good to be killing animals just anyhow and inflicting harm to others or other people."*
2.	NVC	14	17,72	…application of at least one of the four NVC components, or other principles and concepts of NVC	F60: *The way I was before the workshop when I asked something from someone maybe someone will make a joke and say "wait I will give you later" and then for me I would just say " if you don't want you can just stop" - I wouldn't complain. Now it has encouraged me to find a better way to talk to that person so now I know to say if I pose a request to someone I will*

					know the persons needs and my needs so that we are meeting both our needs".
3.	Empowerment	10	12,66	…. a sense of self-improvement or self-determination as a result of the workshops	M47: *For me I see that this programme has changed me with a lot of issues. Now if I'm talking to someone and let's say we have a conflict, the way I was earlier I was short tempered. But this programme has helped me. I do hear a lot of people talk things and there are a lot of things that injure me but now I have a different perspective".*
4.	Animal behaviour	6	7,59	…how participants used new information about how to behave when encountering animals	M41: "*I was walking in a depression where I found some Impalas. The impala were busy grazing and did not notice me. I picked some soil and used it to see the direction of the wind. I noticed the wind was coming from the same direction as I was coming from so I went the other way so they would not detect my scent. I was so close to them and became so interested to see how they are conducting themselves. So I was able to watch them until I got tired. So the person who brought that concept of wind direction, I was able to use that concept"*
5.	Communication skills	2	2,53	… skills people gained that improved their communication skills	M9: *My communication skills have improved. Now at home the way I talk to people it is very much easier for them to understand me. I have lots of friends now. I did not have friends; I had friends but not many. Now big people small people and old people are my friends"*
6.	Fire management	1	1,27	….changes in relation to fire management	M30: After the discussion about volunteering, I organized a team to go and fight a bush fire that started in my area
7.	Accountability	1	1,27	….lack of trust, lack of transparency, lack of applying rules and policies or illegal behaviour	M26: *these lessons actually opened up my mind. Two days ago when I went to visit my cattle I found a trophy elephant dead. Due to these lessons, I didn't tamper with the carcass, maybe to try to get some meat out of it. I just left it like that and went to report to conservancy office. They called MET and went to the carcass and removed the tusks. If I didn't have these lessons, this*

					time around I would've had a good laugh because I would have owned a car by now"
	Total	79	100		

Table S12. Solutions proposed during dialogues

Replace area representatives if they are not communicating back to communities
Ruth do training program for area representatives
Program participants become area representatives
Choose area representatives who can read and write and with proper skills to represent community properly
Create specific criteria to base choice of area representatives
Indunas should represent community as they will distribute meat more fairly
A solution to the lack of information about meeting dates - -when they register new members they should get their phone numbers and sms members
A solution to the lack of information about meeting dates --to have fixed dates throughout year for meetings
A solution to the lack of information about meeting dates -use local radio to communicate about meetings and other conservancy information
Train good communicators who can give empathy to members who have damage from wildlife-this will promote coexistence
MET should have representatives in the villages to allow better communication with community
When payments are being made the field officer should witness to see that correct amount of money is being paid
Appoint community game guard in each village with equipment to record damage as backup for when conservancy game guard cannot/do not come
Appoint game guards who are punctual at work
Work together to find better ways to prevent damages

MET should pay directly to farmer and not go through conservancy as there is more paperwork
Pay double to those who use mm to motivate people to apply mm
Conduct more awareness workshops even inviting government to share ideas on solutions for mm
Use many different methods at same time so animals don't get used to any one method to avoid habituation
To prevent farmers using oil and chilli for other purposes-mix the oil and chilli at office and give to each person
Awareness about animal behaviour and mm
When conservancy get more income from campsites and lodges use money for mm
Locals train and apply to be a professional hunter and get more money instead of outside hunters making all the money
To prevent the professional hunter getting away with not paying, should not give next hunter credit
Consult communities on which policies and how they should be changed
To solve the problem of conservancy not having money to print policy documents for members, members can donate or bring paper to office to make photocopies
NVC training for everyone so that the love and passion will be used to help everyone
Rather than dissolve the conservancy change leadership
Thinking how participants from the program can be involved in cmc- names will be given and recommend to induna to call them for meetings
To solve problem of non-representation at cmc meetings, members of the program should be called for meetings at cmc
To solve problem of negative wildlife impacts, wildlife and people should be separate-each must their have own area
To solve problem of scarcity of wildlife to hunt, national park should give conservancy more land, an extra 20 km.
Humans can show love to animals by treating sick animals. And when sick animals are taken in and treated by people the animals will see that humans are kind and this will reduce HWC
To solve problem of negative wildlife impacts, fence the animals in one area so people could see them when they wanted to
Zonation to separate people, livestock and wildlife

Solution for low value of wildlife in region due to foot and mouth and cattle- process meat and export out of province
To solve problem of negative wildlife impacts, wildlife are fenced in parks. Development happens and we live in houses with running water we don't need to fetch water at river, there are shops so we don't need to farm

Table S13. Record where participants reported sharing information learnt at workshops or an intention to share information.

He shared his knew knowledge and understanding about need for tolerance towards wildlife with aunt who wanted to kill wild dogs after a negative experience
He passed on information and learning to wider community
He plans to meet with community members to spread knowledge learnt from workshops
He shared with other farmers about damage reporting
He is eager to implement awareness and training about mitigation measures before growing season to avoid conflict from annoyed farmers
The discussion from the sculptures made him speak to elders about the history of conservancy and how things were in the past
He was inspired to chat to elders about conservancy issues-they support idea that need good leadership rather than dissolve conservancy
He had discussion about conservancy issue with family and elders
He feels encouraged to share lessons with others, although has not done so yet
He explained about feelings and needs to someone
He shared the lessons from week one with two friends
He has shared information from workshop with others
He taught NVC to his boy and his boy is using it-his boy now asks him how he is feeling
He told his brother to inform conservancy of lion predation despite brother not intending to do so
He intends to report back to CMC on the scenario planning workshops
He intends to invite participants of the workshop to come to CMC meetings
He shared information at meat distribution about constitution-that people must be 18 and register to get benefit

He shares what he has learnt with his mom after each week, others in community always curious to hear what he has learnt
She asked elders their opinions on good and bad things of living with wildlife-elders supported idea of living with wildlife and its benefits such as clothing
He shared learning with family about coexistence as they were watching some wildlife near the village
She shared ideas of empathy, dialogue and tolerance with friends
We had different views during the workshops but we leant from each other and lessons brought us together.
We will tell other community members what we are learning about how the conservancy works and they will be informed to demand change
He is willing to support the enterprise office and come to CMC meetings to share what they have learnt.
